# scPrisma infers, filters and enhances topological signals in single-cell data using spectral template matching

**DOI:** 10.1038/s41587-023-01663-5

**Published:** 2023-02-27

**Authors:** Jonathan Karin, Yonathan Bornfeld, Mor Nitzan

**Affiliations:** 1 School of Computer Science and Engineering, The Hebrew University of Jerusalem, Jerusalem, Israel; 2 Racah Institute of Physics, The Hebrew University of Jerusalem, Jerusalem, Israel; 3 Faculty of Medicine, The Hebrew University of Jerusalem, Jerusalem, Israel

**Keywords:** Computational models, Data processing

## Abstract

Single-cell RNA sequencing has been instrumental in uncovering cellular spatiotemporal context. This task is challenging as cells simultaneously encode multiple, potentially cross-interfering, biological signals. Here we propose scPrisma, a spectral computational method that uses topological priors to decouple, enhance and filter different classes of biological processes in single-cell data, such as periodic and linear signals. We apply scPrisma to the analysis of the cell cycle in HeLa cells, circadian rhythm and spatial zonation in liver lobules, diurnal cycle in *Chlamydomonas* and circadian rhythm in the suprachiasmatic nucleus in the brain. scPrisma can be used to distinguish mixed cellular populations by specific characteristics such as cell type and uncover regulatory networks and cell–cell interactions specific to predefined biological signals, such as the circadian rhythm. We show scPrisma’s flexibility in incorporating prior knowledge, inference of topologically informative genes and generalization to additional diverse templates and systems. scPrisma can be used as a stand-alone workflow for signal analysis and as a prior step for downstream single-cell analysis.

## Abstract (web summary)

scPrisma deconvolutes different biological processes in single-cell RNA-sequencing data.

## Main

In recent years, progress in single-cell RNA sequencing (scRNA-seq) that contains information about the gene expression profiles of the multitude of cells across tissues has led to substantial improvement in our understanding of a variety of intracellular and intercellular processes^1^. Recent computational advances have pushed forward the interpretation of these data to extract information about the heterogeneity of cell types and states and their collective structure and behavior, including spatial context^2^, gene regulatory patterns^3^, cell type^4^ and temporal processes such as lineage^5^ and cell cycle^6^. Because scRNA-seq data contain multiple biological signals, it can be challenging to uncover a particular underlying signal and in many cases, prior information about the signal in question is needed^6,7^. Recently, we have shown how topological priors about the hierarchical structure of lineage and differentiation in single-cell data can be leveraged for their identification using spectral approaches, as they exhibit power-law signatures in the covariance eigenvalue distribution^8^. Relying on such global topological features can provide a more robust and generalizable approach than relying on specific features such as marker genes^6,7^, as these are many times unique to different biological systems and processes and can be challenging to infer for new systems. Here we show how topological priors can be used beyond signal identification. We use priors about the periodicity and linearity of diverse biological processes and later generalize to additional topologies, to either enhance or filter them out from single-cell data using a spectral projection approach.

Biological processes that are inherently periodic are abundant and have important roles in diverse contexts, such as the cell cycle and circadian rhythm. Multiple computational methods aim to infer periodic signals from single-cell data, with a particular focus to extract information related to the cell cycle or to remove its effect^6,7,9,10^. However, the majority of these methods are heavily based on the cell cycle marker gene information, including ccRemover^7^, Seurat^11^ and reCAT^6^, which makes them difficult to generalize across systems and across periodic signals beyond the cell cycle. On the contrary, Cyclum^9^, which does not rely on marker genes, is an auto-encoder-based approach that optimizes a circular embedding for single-cell data to infer and remove cell cycle effects. However, fitting the data to a one-dimensional circle does not generally capture the variability of cyclic biological processes and lacks flexibility as it cannot easily incorporate additional prior knowledge, such as low-resolution temporal information, which may be necessary for weak cyclic signals.

**Fig. 1:**
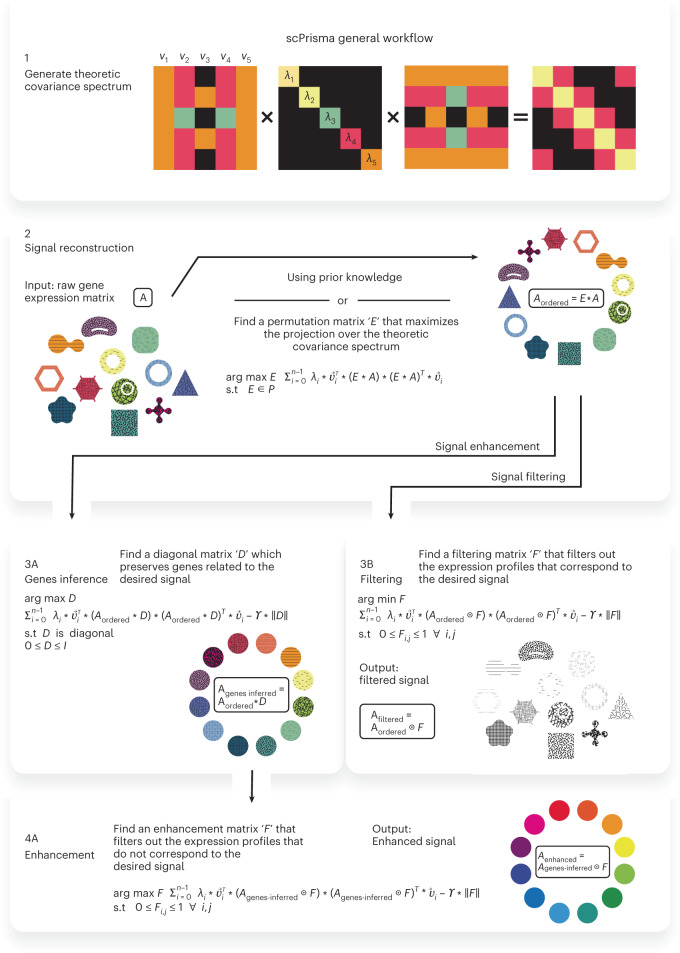
General workflow of scPrisma. Given single-cell data and a theoretical covariance matrix, for example, based on a topological model, we calculate its theoretical spectrum (1), order the cells along the topology (2) and either enhance the signal corresponding to that topology (3A, 4A) or filter it out from the data (3B).

Here we present scPrisma, a general spectral framework (Fig. 1) for the reconstruction, enhancement and filtering of signals in single-cell data based on their topology and inference of topologically informative genes. We benchmark scPrisma and demonstrate its performance over simulated data and seven scRNA-seq datasets. Specifically, we show how the cell cycle can be revealed or filtered in a population of HeLa cells, how circadian rhythm and spatial zonation can be decoupled in liver lobules, how differences in *Chlamydomonas* that were grown in different environments can be emphasized by filtering their diurnal cycle signal, and how the signature of the circadian rhythm can be revealed in multiple cell types in the suprachiasmatic nucleus (SCN) in the brain, the master circadian pacemaker in mammals. In addition, we show how using scPrisma allows us to better distinguish distinct cellular subtypes of SCN neurons following temporal filtering, and uncover signal-related gene regulatory networks and cell–cell interactions following enhancement of the circadian rhythm signal. Finally, beyond cyclic and linear templates, scPrisma can be used to manipulate diverse template types, enhance the separation between clusters, identify multiple cyclic processes and enhance spatial signals in spatial transcriptomics. scPrisma is versatile and enables topological signal manipulation without low-dimensional embedding, which renders the results useful for diverse types of downstream analyses. Furthermore, it is flexible as it enables integration of diverse types of prior knowledge (such as low-resolution temporal ordering) but does not rely on it and can be used for de novo analyses.

## Results

### Spectral template matching for scRNA-seq signal manipulation

**Fig. 2:**
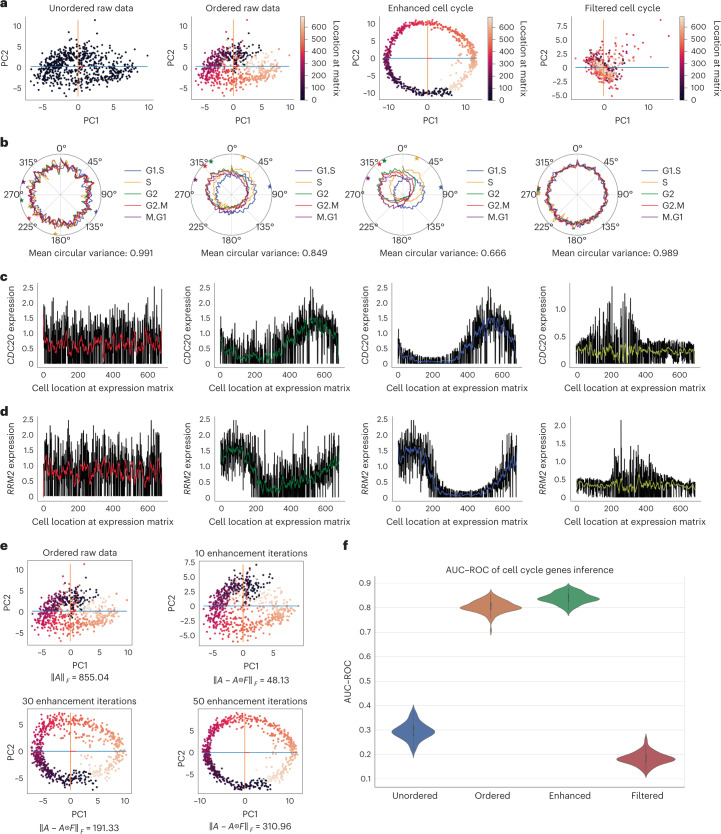
scPrisma manipulates the cell cycle signal in HeLa cells. **a**, PCA representation of 683 cells in the unordered and ordered raw gene expression data, and data following spectral cyclic enhancement and filtering. The cells are colored according to the corresponding rows in the gene expression matrix. **b**, Smoothed polar plot of the normalized sum of the gene sets corresponding to different cell cycle phases^13^. The circular mean of each phase is marked by a correspondingly colored star. **c**,**d**, Expression of *CDC20* (which peaks in the M phase^33^) and *RRM2* (which peaks in the S phase^33^) as a function of cellular location in the gene expression matrix. **e**, PCA representation of cells along iterations of the cyclic enhancement algorithm. **f**, Violin plot of AUC scores for *n* = 50 experiments of sampling random gene subsets and inference of cyclic genes by scPrisma based on unordered, ordered, enhanced and filtered data. White dots mark the median, gray bars mark the interquartile range and thin gray lines mark the rest of the distribution, except for outliers.

**Fig. 3:**
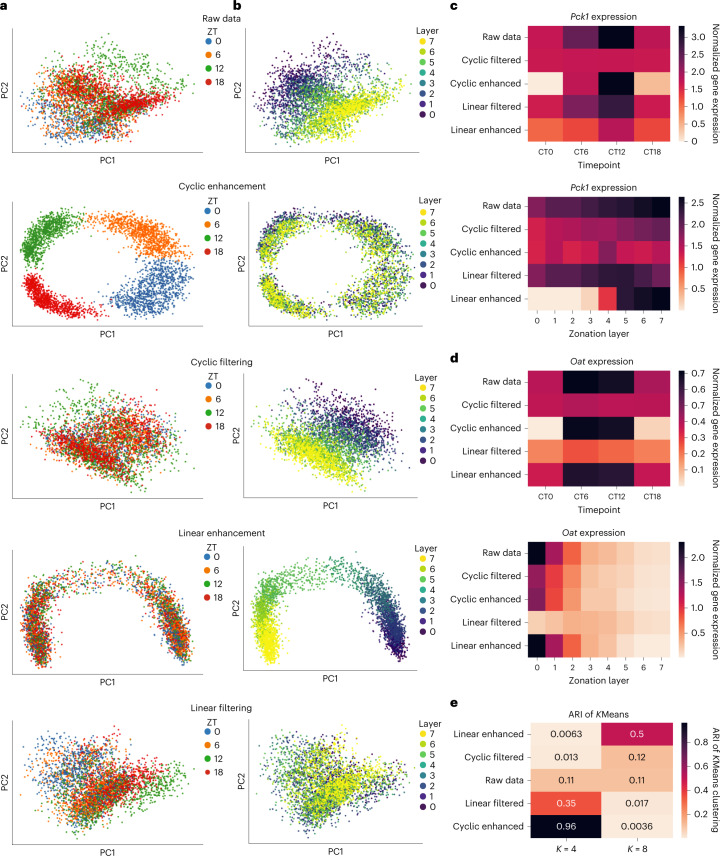
Disentanglement of spatial and temporal signals in liver lobules. **a**,**b**, PCA representation of raw single-cell data of 4,000 cells (1,000 from each time point), and following spectral cyclic enhancement, cyclic filtering, linear enhancement and linear filtering. The cells are colored either according to their associated time points (ZT, sampled at four equally spaced time points along the circadian rhythm) (**a**), or by their respective spatial location (‘layer’, according to the zonation analysis done in ^15^) (**b**). **c**,**d**, Heatmaps of the expression of *Pck1* (**c**) and *Oat* (**d**) following scPrisma analysis as a function of the sampling time and zonation layer. **e**, ARI of *K*Means clustering with *K* = 4 (corresponding to four underlying time points) and *K* = 8 (corresponding to eight underlying zonation layers) after applying each of scPrisma’s spectral algorithms.

**Fig. 4:**
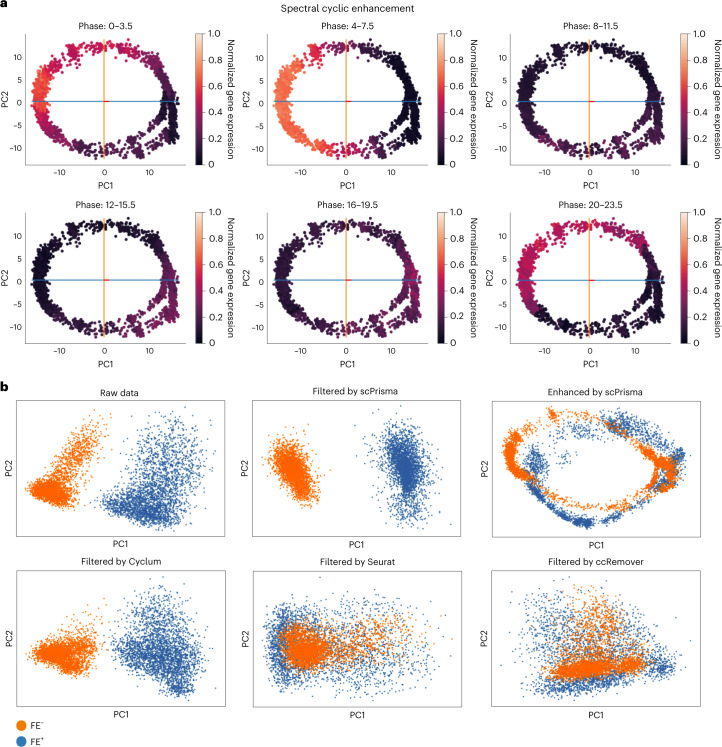
scPrisma detects and filters the diurnal cycle in *Chlamydomonas*. **a**, PCA representation of 3,000 cells in spectrally enhanced cyclic signal of the Fe^+^ experiment^16^. Each plot represents 1/6 of the 24 h cycle. The cells are colored according to the normalized sum of the marker genes associated with each phase^17^. **b**, PCA representation of 6,000 cells (3,000 of each condition) in Fe^+^ (blue) and Fe^−^ (orange) conditions of raw gene expression data, data following scPrisma’s cyclic filtering (Silhouette score before/after filtering = 0.088/0.136, Calinski and Harabasz score before/after filtering = 600.802/1000.44) and enhancement, and data following filtering by Cyclum (Silhouette score = 0.052, Calinski and Harabasz score = 337.629), Seurat (Silhouette score = 9.868 × 10^−^^4^, Calinski and Harabasz score = 1.29 × 10^−^^10^) and ccRemover (Silhouette score = 9.815 × 10^−^^6^, Calinski and Harabasz score = 6.25 × 10^−^^13^).

**Extended Data Fig. 1:**
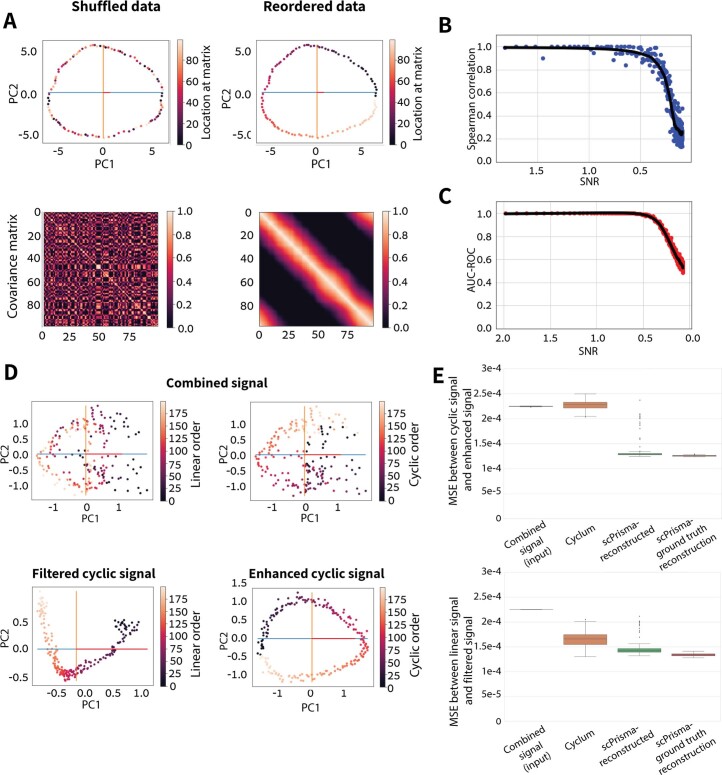
scPrisma identifies and filters periodic signals in simulated data. (A) PCA representation and gene expression covariance matrix before and after applying the reconstruction algorithm. This was done over a simulated gene expression matrix (100 cells, 500 genes, w=0.3) encoding a cyclic signal according to the spatial model (Supplementary A.2). The covariance matrix, after applying the reconstruction algorithm, is circulant. (B) Spearman correlation between the ground-truth permutation and the predicted permutation as a function of SNR over 300 simulations. A cyclic signal was simulated similarly to (A), Gaussian noise was added with varying variance, while the expression matrix was clipped to be positive. (C) AUC-ROC of informative genes inference task as a function of SNR over 300 simulations, each consisting of a cyclic signal and a lineage signal (256 cells, 250 genes) that were concatenated to form a combined gene expression matrix (256 cells, 500 genes), supplemented by additive Gaussian noise. (D) Filtering and enhancement of cyclic signals in combined simulated data. The combined simulation consists of the sum of linear and cyclic signals (200 cells, 500 genes each), and a Gaussian noise matrix (variance = 0.1). (E) Boxplots comparing the filtering and enhancement algorithms of scPrisma with Cyclum over n=100 simulations of the combined signal as described in (D). For the box plots, the center line is the median, box limits are the 0.25 and 0.75 quantiles, vertical lines extend from the top of the box to indicate the maximum value, and from the bottom to indicate the minimum value.

We developed scPrisma, a spectral analysis framework that uses topological priors over underlying signals in single-cell data, to allow for their inference, enhancement and filtering. The core of scPrisma uses spectral template matching between the spectrum (the eigendecomposition of the covariance matrix) of a set of single-cell data (for example, scRNA-seq) and the expected analytical spectrum of a structure or process we aim to enhance or filter. To analyze a theoretical covariance spectrum (by analyzing its eigenvalues and eigenvectors), we need a reference model. Focusing first on cyclic signals, we propose a simple toy model of periodic biological signals (Methods). The covariance matrix of the gene expression matrix of this model is a circulant matrix of a special form that depends on the model parameters (Methods). Circulant matrices have closed-form formula for their eigenvectors and eigenvalues^12^ (Fig. 1–1), which we used to estimate the ordering of cells along the cyclic topology. This was done by optimizing for a permutation matrix that maximizes the projection of the data over the theoretical spectrum (Methods; Fig. 1–2). As input for the remainder of scPrisma’s workflow, cellular ordering can also be informed by prior knowledge of low-resolution pseudotime. Based on the reconstructed ordering, scPrisma infers topologically informative genes, as the set of genes that maximizes the projection over the theoretical spectrum (Methods; Fig. 1–3A). scPrisma can then enhance (filter) the signal related to the cyclic process by filtering out gene expression entries that do not maximize (minimize) the projection over the theoretical spectrum (Methods; Fig. 1–4A and 3B). scPrisma can successfully reconstruct, filter and enhance periodic signals in simulated single-cell data (Supplementary Note A.1, Extended Data Fig. 1 and Supplementary Fig. 1).

### scPrisma manipulates the cell cycle signal in HeLa cells

We first tested our approach on a scRNA-seq dataset of HeLa cells, unsynchronized across the cell cycle^13^ (Fig. 2). To assess the results, we used a list of approximately 400 genes, classified according to cell cycle phases^13^, where for each phase, the corresponding genes were summed and normalized and their circular mean and variance were calculated^14^ (Supplementary Note A.4). For an ordered reconstructed signal, the ordering of the circular means should correspond to the cell cycle phases and the circular variance should be less than 1, while for randomly ordered data, the circular variance is expected to be close to 1 (corresponding to a uniform signal along the cycle). Following standard preprocessing of the HeLa single-cell data (Methods; Supplementary Note A.4), the distributions of all phases were found to be nearly uniform (Fig. 2b; mean circular variance = 0.991). However, after cyclic ordering by scPrisma (Methods), different phases of the cell cycle became clearly separated (Fig. 2b; mean circular variance = 0.849) and peaked progressively according to the correct phase ordering.

To further enhance the cell cycle signal in the data, we employed scPrisma’s cyclic enhancement algorithm. Iterations of our algorithm gradually revealed a cyclic signal, which was apparent after filtering less than 7% of the total signal and was clearly revealed after removing 22% of the total signal (Fig. 2e). In addition, the reconstructed angular ordering was correlated with the cell cycle phases and the circular variance of phase-corresponding marker genes decreased substantially (Fig. 2b; mean circular variance = 0.666). Further, genes associated with different cell cycle phases peaked progressively in their expected order (Methods; Fig. 2c,d and Supplementary Fig. 2).

Next, we approached the reverse challenge: to filter out the cell cycle signal from the HeLa cells data. After applying scPrisma’s cyclic filtering algorithm to the ordered data, the separation between different cell cycle phases and their corresponding progressive peaks was lost (Fig. 2b; mean circular variance = 0.989).

Finally, we identified genes related to the cell cycle using the genes inference algorithm (Methods; Supplementary Note A.4). When using as input both randomly selected subsets of cell cycle-related genes^13^ and subsets of genes unrelated to the cell cycle, we found a substantial improvement in our ability to identify cell cycle-related genes after reordering the cells (mean AUC = 0.804) relative to the original data (mean AUC = 0.295). Moreover, relative to the ordered data, identifying cell cycle-related genes was further improved following spectral cyclic enhancement (AUC mean 0.838, outperforming available baselines; Supplementary Fig. 2) and was diminished following cyclic filtering (mean AUC 0.183; Fig. 2f).

### scPrisma disentangles spatiotemporal signals in the liver

We next dissected spatiotemporal signals via a scRNA-seq dataset which captures gene expression variation of hepatocytes in the mammalian liver across both space (spatial zonation across the periportal to pericentral axis) and time (temporal variation across the circadian rhythm)^15^. Similarly to the cell cycle, the circadian rhythm was also expected to exhibit a cyclic structure in gene expression space. Here we used experimental prior knowledge in the form of low-resolution sampling time^15^ to order the cells in a cycle. In this setting, we still missed information about temporally informative genes, and spatiotemporal information was still entangled (for example, *Pck1* varied informatively across both space and time of day^15^). We leveraged scPrisma to disentangle these data and showed clear enhancement and filtering of the circadian rhythm, relative to raw data (*K* = 4 Adjusted Rand Score (ARI) of *K*Means = 0.96; 0.013; 0.11, respectively; Fig. 3a,e and Supplementary Fig. 3A; Methods). scPrisma outperformed available baselines for filtering the circadian rhythm (Supplementary Note A.5). These results were reflected in the behavior of individual genes; *Pck1* is a rhythmic gene that was highly expressed at ZT06 and ZT12 (ref. ^15^), and indeed, following spectral cyclic enhancement, its resulting expression in ZT00 and ZT18 was diminished, while cyclic filtering resulted in a nearly constant temporal expression of *Pck1* (Fig. 3c). Spatially, *Pck1* is periportally zonated, and indeed, spectral cyclic enhancement flattens its spatial expression, while cyclic filtering retains its spatial variation (Fig. 3c). Similar behavior can be observed for additional spatiotemporally informative genes (Fig. 3d and Supplementary Fig. 4).

In a complementary manner, spectral analysis focusing on the characteristics of linear signals can be used to filter the spatial linear signal in the collective gene expression of hepatocytes (Methods). Indeed, following spectral linear filtering by scPrisma, the cyclic circadian signal was clearly revealed (Fig. 3a and Supplementary Fig. 3A) and the linear zonation signal was blurred out relative to the raw data (Fig. 3b and Supplementary Fig. 3B,E; *K* = 8 ARI of *K*Means = 0.017; 0.11, respectively). Focusing again on *Pck1* expression, linear spectral enhancement retained only the expression around the portal vein and reduced the temporal variance, while linear filtering reduced the zonation variance and yet retained the temporal variance (Fig. 3c).

Finally, we evaluated the dominant structure in the data following scPrisma analysis. As predicted, following spectral enhancement, the data clustered according to the enhanced signal, while following spectral filtering, the data clustered according to the unfiltered signal; for example, filtering the cyclic signal led to better identification and clustering of the spatial zonation signal (Fig. 3e).

### scPrisma manipulates the diurnal cycle in *Chlamydomonas*

To demonstrate the use of scPrisma for more complex systems with diverse prior knowledge, we next turn our attention to scRNA-seq data collected for *Chlamydomonas* (green algae), grown under two contrasting conditions, iron replete (Fe^+^) and iron deficient (Fe^−^)^16^. In both conditions, an expression signal that reflects the 24 h diurnal cycle was previously detected^16^. To evaluate progression of cells along the diurnal cycle, we used marker genes corresponding to different cycle phases obtained from bulk RNA-sequencing^17^ (Supplementary Note A.6). Using scPrisma’s cyclic enhancement resulted in robust reconstruction of the diurnal cycle for each of the two conditions. This was done by splitting the 24-h cycle into six phases and validating that they are well separated following reconstruction and enhancement (Methods; Fig. 4a for Fe^+^ condition and Supplementary Fig. 5A for Fe^−^ condition). Further, concatenating the enhanced cyclic signal of both experiments resulted in a reconstructed synchronized diurnal cycle (Supplementary Fig. 5B). We next focused on enhancing the biological differences between the Fe^−^ and Fe^+^ conditions by spectrally filtering their shared diurnal cycle (Fig. 4). As expected, cyclic filtering increased the differences between the clusters of Fe^−^ and Fe^+^ associated cells (Silhouette score before/after filtering = 0.088/0.136, Fig. 4b). scPrisma outperformed state-of-the-art cyclic filtering methods, including ccRemover^7^, Seurat^10^ and Cyclum^9^ (Silhouette scores = 9.815 × 10^-6^, 9.868 × 10^-4^ and 0.052, respectively; Supplementary Note A.8 and Fig. 4b).

### scPrisma extracts SCN cell-type-specific temporal signals

**Fig. 5:**
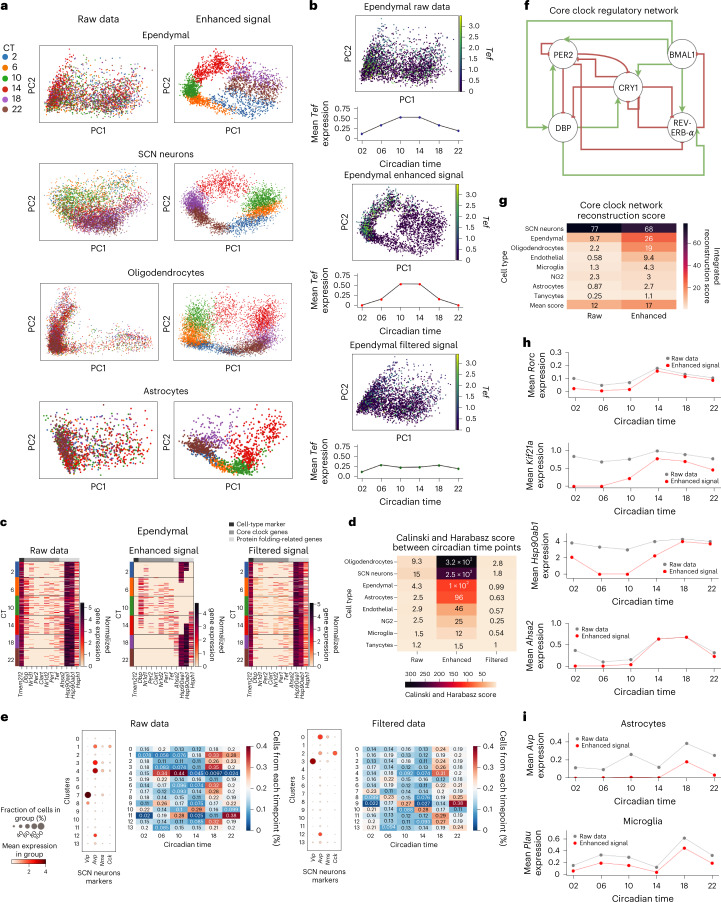
scPrisma extracts cell-type specific circadian rhythm signals in the SCN. **a**, PCA representation of cells in raw SCN gene expression data^18^ and data following cyclic enhancement by scPrisma, colored according to CT, for several cell types: ependymal, SCN neurons, oligodendrocytes and astrocytes. **b**, PCA representation of ependymal cells colored by *Tef* expression and mean *Tef* expression as a function of circadian time, for raw data (top), data following scPrisma’s cyclic enhancement (middle) and filtering (bottom). **c**, Heatmaps of ependymal expression of (from left to right) cell-type marker genes, rhythmic genes and protein folding genes, for raw data (left) and data following scPrisma’s cyclic enhancement (middle) and filtering (right). **d**, Calinski and Harabasz score for cells sampled at different CT for raw data, and data following scPrisma’s cyclic enhancement and filtering. Cell types with low fraction of circadian genes^18^ exhibit lower scores. **e**, Pre- and post-filtering gene expression dotplots of the marker genes of SCN neurons (before filtering, clusters 1, 3 and 4 contain mixtures of two different subtypes), and pre- and post-filtering heatmaps showing the contribution of cells sampled at different CT to each neuronal cluster. **f**, Regulatory network structure of core clock genes^21^. **g**, Pre- and post-enhancement sum of scores of all regulatory interactions of the core clock network shown in **f**, scored by GRNBoost2, for each SCN cell type. **h**, Pre- and post-enhancement mean *Rorc*, *Ahsa2* and *Hsp90ab1* expression as a function of CT. Following cyclic enhancement, regulatory interactions between the transcription factor *Rorc* and *Ahsa2*, *Hsp90ab1* were uncovered. **i**, Mean *Avp* and *Plau* expression as a function of CT. Following cyclic enhancement, cell–cell interactions between astrocytes and microglia cell types, mediated by *Avp* and *Plau*, were revealed.

We next focus on scRNA-seq data collected for mice SCN, the mammalian brain’s circadian pacemaker^18^. In this experiment, cells were sampled at 12 time points along two days. Again, we leveraged the cyclic nature of the circadian rhythm and explicitly used the prior knowledge regarding the experimental sampling times (instead of running the reconstruction algorithm). We first clustered the cells using the Louvain algorithm and mapped individual clusters to cell types using established marker genes^18^ (Supplementary Note A.7 and Supplementary Fig. 6). scPrisma’s cyclic enhancement over each cell type separately revealed a cyclic signal associated with the circadian rhythm for 5/8 of cell types (Fig. 5 and Supplementary Fig. 7; Methods). The three cell types that did not expose a clear cyclic signal (NG2, microglia and tanycytes) exhibit the lowest fraction of rhytmic gene expression^18^. Moreover, we measured the separation of cells that were sampled at different time points, before and after cyclic filtering/enhancement using the Calinski and Harabasz score^19^. Overall, as expected, separation increased substantially following cyclic enhancement and decreased following cyclic filtering, which, as above, is least substantial for the three cell types exhibiting the lowest fraction of rhythmic genes (Fig. 5d). It can be observed that the cellular density varies with the rhythmic process and is correlated with the peak of temporal expression of rhythmic genes (Fig. 5a,b and Supplementary Note A.7). Focusing on gene expression, we found that spectral cyclic enhancement diminishes the expression of cell-type marker genes and retains the expression of rhythmic genes (core clock genes and protein folding genes, as characterized in ref. ^18^; Fig. 5c and Supplementary Fig. 7). Conversely, following cyclic filtering, cell-type marker gene expression was retained, while the resulting temporal expression of rhythmic and protein folding-related genes flattened (Fig. 5c and Supplementary Fig. 7).

**Extended Data Fig. 2:**
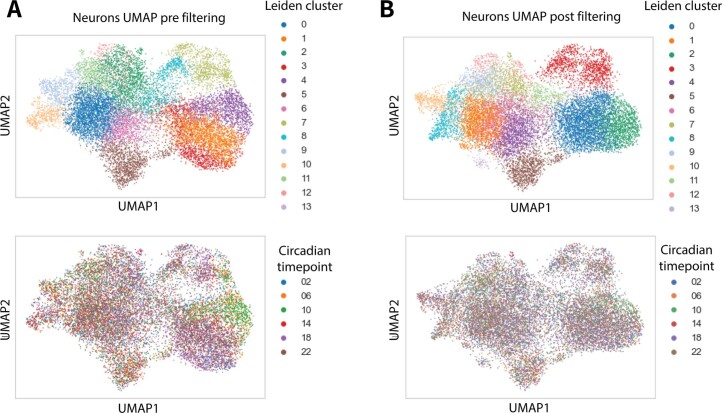
Temporal filtering by scPrisma enhances cell type clustering of single-cell data. Analysis based on single-cell data of suprachiasmatic nucleus neurons^15^. (A) Before temporal filtering, clusters ‘1’ and ‘3’ mostly contain cells that were sampled in CT=14/18/22 while cluster ‘4’ mostly contains cells that were sampled in CT=06/10. (B) However, following filtering, the corresponding clusters (now labeled ‘0’ and ‘2’) are well-mixed in terms of their temporal labels.

scPrisma further enhanced cell-type classification, inference of gene regulatory interactions related to the circadian rhythm and underlying cell–cell interactions. When aiming to characterize cells by their type, additional biological signals can interfere with that task as similarity between cells can arise due to multiple factors. For example, direct clustering of cells according to their gene expression profiles may capture similarities according to the circadian rhythm phase and not their type, which can substantially hinder our ability to distinguish different cell types. Clustering the neurons yielded 14 distinct clusters, three of which can be identified using either established marker genes or previous subtype classification^18^ as containing mixture of neurons from both SCN neuronal subtypes N0 and N2 (clusters 1, 3, 4; Supplementary Note A.7 and Fig. 5e). We found that the circadian rhythm signal interferes with the proper classification of cell subtypes in this case, supported by the observation that in clusters 1 and 3 the majority of cells (79% and 66%, respectively) were sampled at circadian time points (CT) = 14/18/22, while in cluster 4, 93% of cells were sampled at CT 02/06/10, which suggests that the clustering of cells in this subpopulation is dominated by their distinct temporal signatures and not their types (Fig. 5e and Extended Data Fig. 2). We were able to overcome the cell-type misclassification by spectrally filtering the circadian rhythm signal using scPrisma, after which, the clustering algorithm yielded a unique cluster for each of the two neuronal subtypes, N0 and N2 (Fig. 5e). Moreover, as expected, the distribution over CT within each cluster flattened following cyclic filtering (Fig. 5e and Extended Data Fig. 2; mean circular variance increased from 0.781 to 0.863 following filtering).

Mixed biological signals in single-cell data can also interfere with the inference of gene regulatory networks. Therefore, we used scPrisma to highlight a set of regulatory interactions related to the circadian rhythm that were difficult to identify in the original data. Specifically, we expected that regulatory interactions that can be revealed following cyclic enhancement would be enriched with interactions associated with the cyclic circadian process. Indeed, regulatory interactions between core clock genes, as inferred using the gene regulatory network inference algorithm GRNBoost2 (ref. ^20^), are more highly correlated to the established core clock interaction network^21^ (Fig. 5f) in the cyclically enhanced single-cell data, relative to the raw data, for 7/8 of the cell types (Fig. 5g and Supplementary Note A.7). Going beyond the core clock interaction network, using a list of known mice transcription factors^22^, we searched for inferred interactions (based on GRNBoost2) which are substantially enhanced following scPrisma cyclic analysis (Methods), where the regulator is a core clock transcription factor (*Nr1d1*, *Nr1d2*, *Rora*, *Rorb*, *Rorc*, *Dbp*, *Tef*^23^; a full list of inferred interactions is available in Supplementary Table 1). For example, focusing on genes inferred to be highly regulated by *Rorc* in ependymal cells following spectral enhancement, we found that the genes that received the highest score were *Ahsa2* (0 to 6.341), *Kif21a* (0 to 6.231), *Hsp90ab1* (0.070 to 5.731) and *Mt1* (0 to 5.002) and the peaks of these genes along the circadian rhythm overlapped with the peak of *Rorc*, following spectral enhancement (Fig. 5h and Supplementary Fig. 7E). These results are consistent with previous results showing the existence of a regulatory interaction between *Rorc* and *Hsp90ab1* which is dependent on the time of day^24^.

Finally, we used scPrisma to infer hidden cell–cell interactions related to the circadian rhythm. We compared cell–cell communication patterns, using CellPhoneDB^25^, between different cell types at corresponding time points. Similarly to the regulatory network inference described above, we were able to recover interactions that were substantially enhanced following scPrisma’s cyclic analysis (Methods; Fig. 5I, Supplementary Note A.7 and Supplementary Table 2).

### Generalized template matching by scPrisma

**Extended Data Fig. 3:**
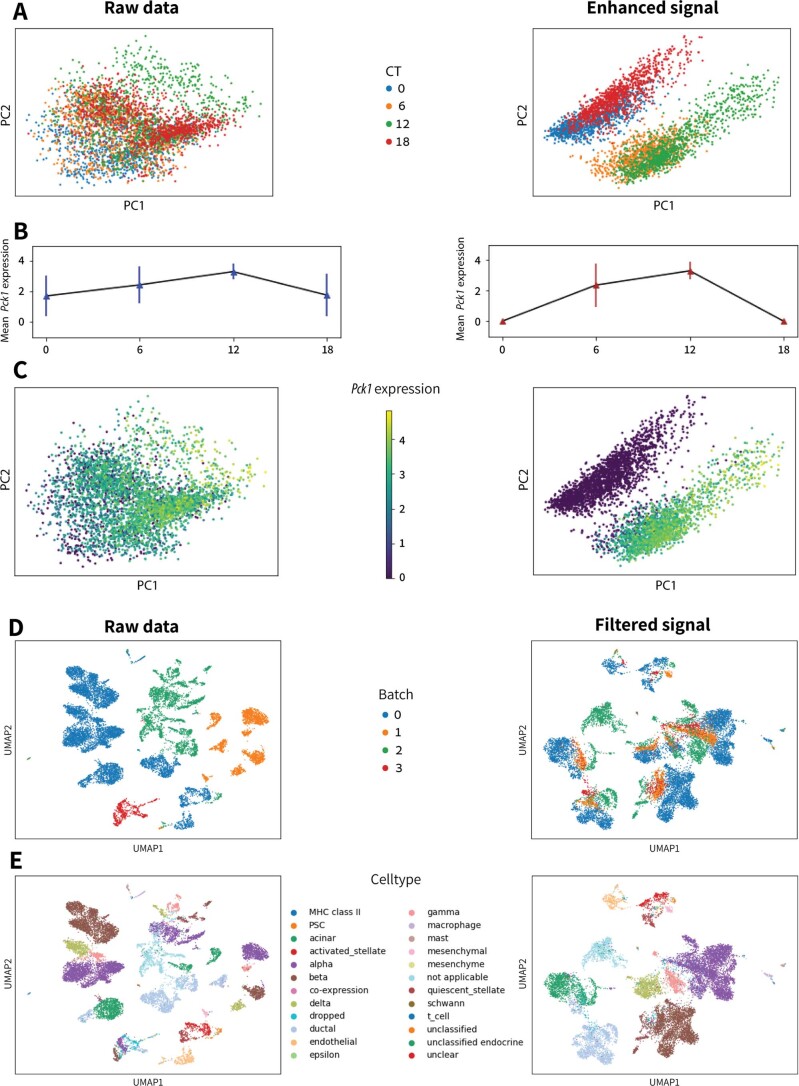
Enhancement and filtering by scPrisma of clustered structure in cellular populations. (A-C) Enhancement of clustered structure in the liver lobule scRNA-seq data^15^; (A) 2D PCA of raw (left) and cluster-based enhanced (right) data, highlighting the separation between samples that were collected during the night (ZT = 0, 18) and the day (ZT = 6, 12). (B) Mean (triangle) and variance (error bar) of *Pck1* expression as a function of circadian time, for raw (left) and cluster-based enhanced (right) data. At each time point, the sample size is N=1000. (C) 2D PCA colored by *Pck1* expression for raw (left) and cluster-based enhanced (right) data. (D,E) Filtering of clustered structure, leading to integration of pancreas scRNA-seq data collected from 4 different studies^26–29^; UMAPs of pre and post data integration, colored by (D) batch and (E) cell type.

**Extended Data Fig. 4:**
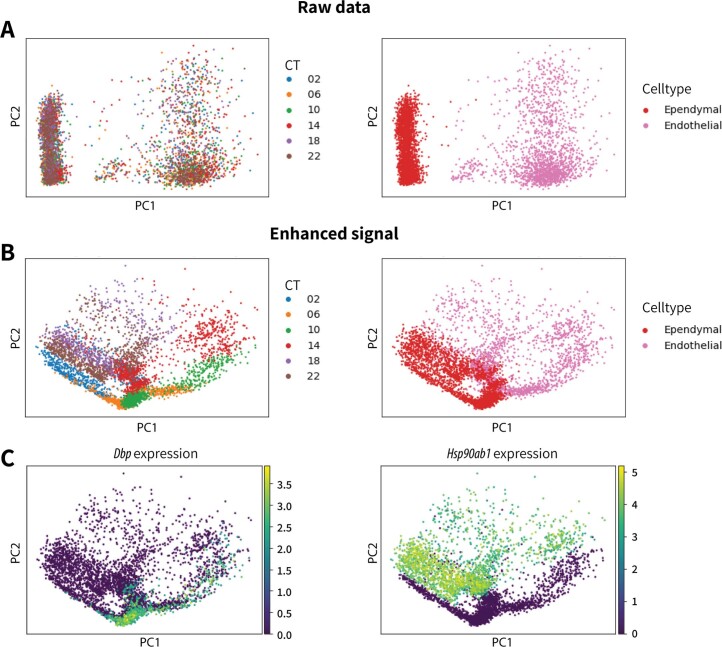
Enhancement by scPrisma of synchronized periodic processes. Analysis based on SCN single-cell gene expression data^18^. (A-C) 2D PCA of ependymal and endothelial cells, for (A) raw and (B,C) cyclic-enhanced data, colored by (A,B) circadian sample time (left) and by cell type (right), and by (C) *Dbp* (left) and *Hsp90ab1* (right) expression.

**Extended Data Fig. 5:**
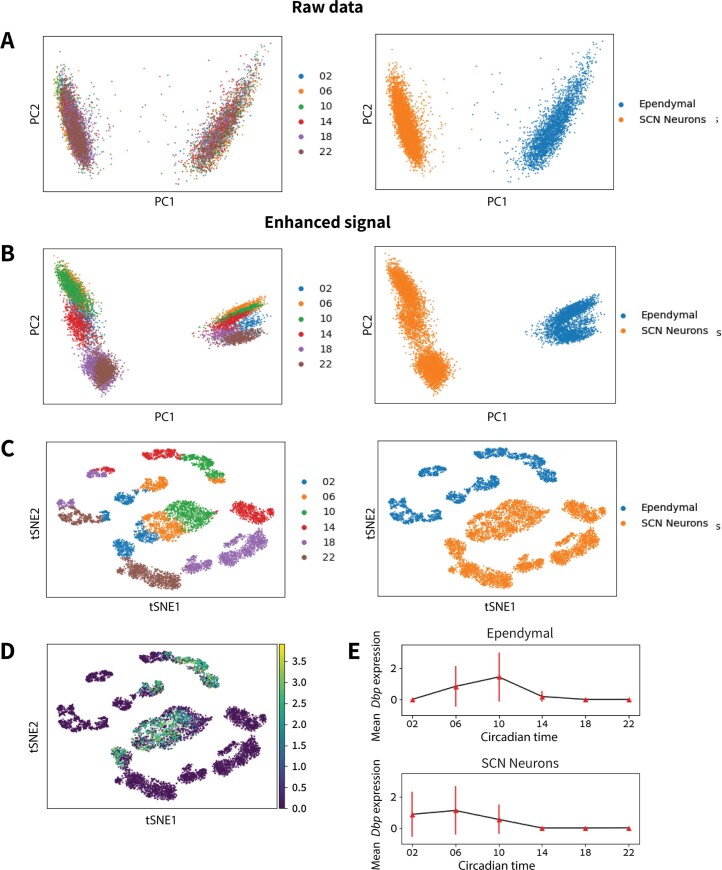
Enhancement by scPrisma of unsynchronized periodic processes. Analysis based on SCN single-cell gene expression data^18^. (A,B) 2D PCA of ependymal cells and SCN neurons, for (A) raw and (B) cyclic-enhanced data, colored by circadian sample time (left) and by cell type (right). (C,D) 2D tSNE of ependymal cells and SCN neurons, for cyclic-enhanced data, colored by circadian sample time (C; left), cell type (C; right), and *Dbp* expression (D). (E) Mean (triangle) and variance (error bar) of *Dbp* expression as a function of circadian time, which peaks at CT = 10 in ependymal cells (top), and peaks at CT = 2/6 in SCN neurons (bottom). The sample sizes at each time point (CT/N) for ependymal cells are: CT02/428, CT06/493, CT10/521, CT14/519, CT18/365 and CT22/480, and for SCN neurons they are: CT02/667, CT06/741, CT10/870, CT14/670, CT18/1375 and CT22/1029.

**Extended Data Fig. 6:**
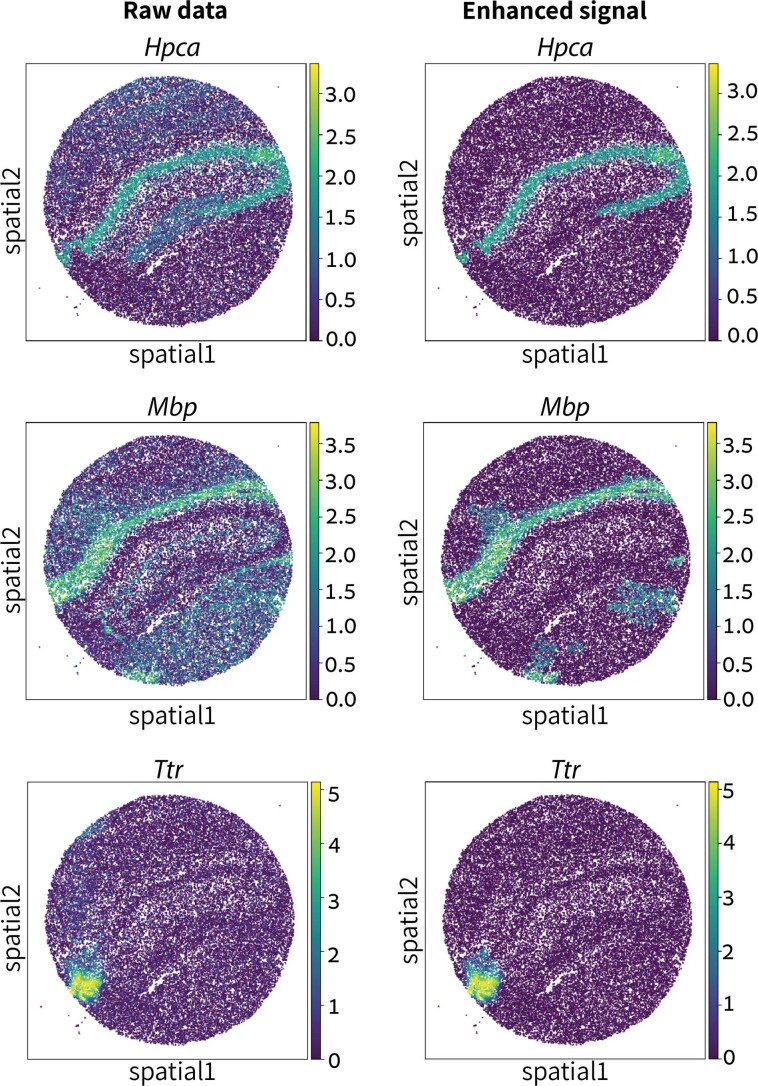
Spatial enhancement by scPrisma. Analysis based on Slide-seqV2 mouse hippocampus data ^32^(subsampled to 29,250 cells). Raw (left) and spatially-enhanced (right) gene expression of the top 3 spatially informative genes (based on Moran’s I score).

Beyond cyclic and linear topologies, scPrisma can be used to manipulate a variety of different, complex topological signals in single-cell data. This is possible because scPrisma can use the numerical spectrum of a given covariance matrix, instead of the analytical spectrum, as was the case for the cyclic and linear topologies. We demonstrated the diversity of scPrisma on three additional types of templates as follows: (1) Clusters—we constructed a cluster-based template to enhance the separation, or maximize the variation in gene expression, between different cellular clusters and states (Supplementary Note A.13), specifically between cellular states of hepatocytes during day and night time (Extended Data Fig. 3A). Further, this topology can be used for data integration via the spectral filtering workflow, as we demonstrated for human pancreas scRNA-seq data which were collected from four different studies^26–29^, labeled and preprocessed as given in ref. ^30^. scPrisma can filter batch effects in this case, demonstrated visually (Extended Data Fig. 3D,E) and quantitatively, as following cluster-based filtering, the Calinski and Harabasz score between the different batches drops from 298.63 to 3.80 and the score between the annotated cell types rises from 90.97 to 107.48. scPrisma’s results for batch correction are competitive or outperform state-of-the-art tailored methods for this task (Supplementary Note A.13). (2) Multiple cycling processes—scPrisma can reconstruct multiple cycling processes across SCN cell types both for a synchronized case (Extended Data Fig. 4) and an un-synchronized case (Supplementary Note A.13 and Extended Data Fig. 5). In the latter, more challenging case, circadian core clock genes are out-of-phase in the SCN neurons versus multiple other cell types^18^. scPrisma can enhance every periodic signal separately by designing a covariance matrix that is block circulant (Supplementary Note A.13). Furthermore, scPrisma can be used for the general, iterative inference of cyclic processes, although this task is more challenging and scPrisma is not optimized for it. As an example, we used human embryonic stem cells (hESC) single-cell dataset^31^. We showed that scPrisma can be used (de novo, without prior knowledge on marker genes) to first infer a cyclic process corresponding to the cell cycle and then filter it out and use the filtered data to reconstruct a second cyclic process corresponding to an oscillatory pattern related to the experimental setup (Supplementary Note A.13 and Supplementary Fig. 8). The encoding of both oscillatory processes by the hESC population is consistent with previous findings of ref. ^31^. (3) Two-dimensional (2D) tissue organization—last, we enhanced the spatial signal in a spatially informed (Slide-seqV2) 2D dataset of the mouse hippocampus^32^. We computed the shortest path matrix (calculated based on the spatial k-nearest neighbor graph over the data) and transformed it into an affinity matrix using a heat kernel (Supplementary Note A.13). In this case, the affinity matrix is used by scPrisma as the covariance matrix of the spatial signal. scPrisma enables flexible manipulation of the spatial signal, which we leveraged to extract, and then either enhance or filter the spatial signal in the data by applying the enhancement algorithm based on numerical eigendecompositions of the affinity matrix (Supplementary Note A.13 and Extended Data Fig. 6).

## Discussion

In this study, we developed scPrisma, a spectral analysis workflow based on topological priors for reconstruction, informative genes inference, signal filtering and enhancement. While we focus on periodic signals (cell cycle in HeLa cells, diurnal cycle in *Chlamydomonas* and circadian rhythm in the SCN), we also demonstrate it for convoluted spatial and cyclic signals (spatial zonation and circadian rhythm in liver lobules) and a diversity of additional signals and topologies, such as clusters, multiple cycling processes and 2D spatial templates.

scPrisma presents three major contributions as follows: First, it embodies a full workflow for analyzing underlying topological signals based on an approach that can be performed either de novo or enhanced using prior knowledge (for example, low-resolution pseudotime or marker gene information). This flexibility allows scPrisma to uncover topological signals of varying strengths. Second, scPrisma enables both signal enhancement and filtering without embedding to lower dimensions, which makes it useful as a prior step for existing downstream analyses, such as inferring gene regulation networks and cell–cell interactions. This can accelerate biological discovery, as we exemplify for SCN neurons, by revealing gene regulation patterns and cell–cell interactions that are associated with a specific biological process such as the circadian rhythm. Third, the enhancement algorithm does not overfit to a circular topology, by applying the genes inference task before enhancement (thus retaining only genes related to the desired signal), controlling the level of filtering by regularization and restricting the range of entries in the filtering matrix. Future work can leverage scPrisma’s flexibility and robustness to optimize it to diverse tasks that arise in the context of single-cell and spatial omics analysis, such as generalized spatial analysis, data integration and iterative manipulation of signals, which is a promising, yet challenging, direction for future work.

**Extended Data Fig. 7:**
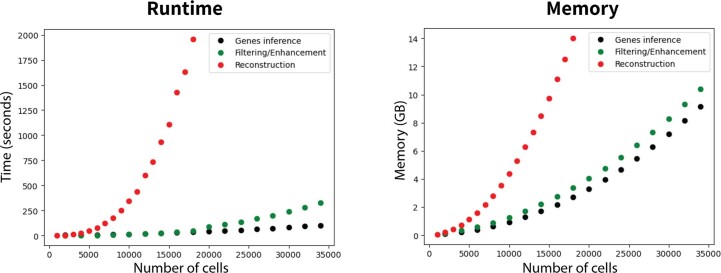
Runtime and memory consumption analysis of scPrisma algorithms. Simulated cyclic signal of 2000 genes, varying number of cells, *w* = 0.3 and added Gaussian noise (variance=0.1), using a single GPU-‘NVIDIA RTX A5000’.

A computational challenge arises due to the nonconvexity and runtime complexity of the reconstruction task (Extended Data Fig. 7). This optimization challenge is relieved for cyclic signals, as the theoretical analysis of the eigenvectors does not depend on the specific values of the matrix but only on its circulant property. Moreover, the multiple solutions for the cyclic reconstruction task (every circular shift of a solution is a valid solution) ease the convergence to a feasible solution. In addition, the reconstruction step can be done either by scPrisma or by other pseudotime trajectory reconstruction algorithms. Another challenge is applying scPrisma when it is not clear whether a signal corresponding to the template exists or is strong enough to be detected in the data. This challenge is alleviated due to several reasons. First, we reason and provide empirical support on both synthetic and real single-cell data that scPrisma avoids overfitting to input template topologies. Therefore, in general, scPrisma does not converge to a topology that is not a reflection of a strong-enough signal in the data, as is demonstrated for the SCN, where the algorithm is limited in its convergence over cell types with weak periodic signal. Second, evaluating results obtained by scPrisma can be done by comparison to partial prior knowledge, such as marker genes known to be related (or unrelated) to recovered biological signals, low-resolution sampling times of cells relative to temporal signals, or by interpreting gene ontology enrichment analysis following signal manipulation. Additionally, we suggest a measure for the quality of convergence of scPrisma, the projection proportion score, and while it can be useful for exploring analysis options, it is not associated with a single threshold that can distinguish successful convergence, as it is affected by the signal and data characteristics (Supplementary Note A.12 and Supplementary Fig. 9).

While in this work we focused on periodic signals, which can be analyzed analytically, scPrisma can also be applied using a numerical eigendecomposition of a covariance matrix that is either inferred from the data or constructed numerically based on a topological model. We anticipate that scPrisma will accelerate single-cell-based research by enhancing target signals of interest and enabling their identification and analysis and providing a general workflow for single-cell signal disentanglement in diverse biological contexts.

## Methods

### Spectral analysis of cyclic signals

For theoretical analysis, we constructed three simple models for the cyclic signals. In the first model, illustrated in Supplementary Fig. 10, we receive as input the number of cellular variations (*q*), the numbers of genes (*p*) and the number of changes between neighboring cells (*k*). We start with a root cell whose expression profile is a binary vector with *p* entries ({1, 0}^*p*^). Each gene is approximated to be either expressed (ON,1) or not expressed (OFF,0). Then, the next cell in the cycle is generated by duplicating the existing cell, choosing uniformly *k* genes and switching their state. This process is repeated *q* times. Then, within the last generated cell, *k* genes whose state differs from the root cell are chosen at random, their state is switched and the cell is duplicated. This process is then continued until the gene expression of the newest cell is identical to the root cell. For the analysis of the covariance matrix of the model, we will use a similar Markovian assumption to the assumption that was used in refs. ^8,34^; the covariance between the expression profiles of two cells, separated by *m* state changes, where *m* is the minimum distance between the cells clockwise and counterclockwise (undirected cyclostationary assumption), is given by $\alpha (m)=E[X(m)X(0)]=\exp (-2mk/p),$ where 0 ≤ *m* ≤ *n*/2, *p* is the number of genes and *k* is the number of changes between neighboring cells. More information about the model and the estimation of *α* from real data is described in Supplementary Note A.11. According to this assumption, the expected covariance matrix of the gene expression matrix is circulant:

1 $$\frac{1}{n}E[X{X}^{\top }]=\left(\begin{array}{llllll}1&\alpha &{\alpha }^{2}&\ldots &{\alpha }^{2}&\alpha \\ \alpha &1&\alpha &\ldots &{\alpha }^{3}&{\alpha }^{2}\\ \vdots &\vdots &\vdots &\ldots &\vdots &\vdots \\ {\alpha }^{2}&{\alpha }^{3}&{\alpha }^{4}&\ldots &1&\alpha \\ \alpha &{\alpha }^{2}&{\alpha }^{3}&\ldots &\alpha &1\end{array}\right)$$

where *n* is the number of cells. The first column ($\overrightarrow{c}$) of a circulant matrix specifies the entire matrix. The (*k*, *j*) entry of a general circulant matrix *C* is given by ${C}_{k,j}={\overrightarrow{c}}_{(j-k) \% n}$^35^. The spectrum of a circulant matrix has analytical closed formula^12^. Specifically, the eigenvalues are the discrete Fourier transform of the first row, and the eigenvectors are the normalized Fourier modes. Because a covariance matrix is symmetric and positive semidefinite, all its eigenvalues are real. Therefore, the eigenvalues are the discrete cosine transform of the first row^36^:

2 $${\lambda }_{i}=\mathop{\sum }\limits_{j=0}^{n/2-1}{{\alpha }\,^{j}}_{i}* \cos \left(\frac{2\pi ji}{n}\right)\,+\,\mathop{\sum }\limits_{j=n/2}^{n-1}{{\alpha }\,^{j}}_{n-i}* \cos \left(\frac{2\pi ji}{n}\right)$$

The *s*th entry of the *i*th eigenvector corresponding to the *i*th eigenvalue is^35^

3 $${q}_{i}=\sqrt{\frac{2}{n}}* \cos \left(\frac{2\pi is}{n}-\frac{\pi }{4}\right)$$

To test our approach, we defined two additional models, described in Supplementary Note A.2, which we used in the simulated data section (Supplementary Note A.1).

### Spectral analysis of linear signals

Similarly to the analysis of cyclic signals, we first construct a simple model for linear signals. We follow a similar linear model to the one that was presented in ref. ^8^. The model receives the same input as the cyclic model, and each cell is represented by a binary vector {1, 0}^*p*^. We start from a root cell, and then over *n* iterations, a new cell is created in the linear chain by changing the state of *k* randomly chosen genes relative to the previous cell in the chain. As in the cyclic model, we assume that the covariance between the gene expression profiles of two cells, separated by *m* state changes, is given by $\alpha (m)=E[X(m)X(0)]=\exp (-2m/p)$. Thus, the expected covariance matrix of the gene expression matrix is

4 $$\frac{1}{n}E[X{X}^{\top }]=\left(\begin{array}{llllll}1&\alpha &{\alpha }^{2}&\ldots &{\alpha }^{n-2}&{\alpha }^{n-1}\\ \alpha &1&\alpha &\ldots &{\alpha }^{n-3}&{\alpha }^{n-2}\\ \vdots &\vdots &\vdots &\ldots &\vdots &\vdots \\ {\alpha }^{n-2}&{\alpha }^{n-3}&{\alpha }^{n-4}&\ldots &1&\alpha \\ {\alpha }^{n-1}&{\alpha }^{n-2}&{\alpha }^{n-3}&\ldots &\alpha &1\end{array}\right)$$

This matrix is a special case of a Toeplitz matrix and is particularly known as Kac–Murdock–Szego matrix^37^. The eigenvalues of such a Kac–Murdock–Szego matrix can be approximated as ref. ^37^:

5 $${\lambda }_{i}=\frac{1-{\alpha }^{2}}{1+{\alpha }^{2}-2{\mathrm{cos}}\left(\frac{\left(i+1\right)\pi }{n+1}\right)\alpha }$$

The corresponding eigenvectors can either be estimated analytically^38^ or calculated by the numerical decomposition of the theoretical matrix.

### Preprocessing

We used a standard preprocessing pipeline as follows: first removing genes that are not expressed in any of the cells in our data, applying per-cell normalization by dividing each count by the total counts of that particular cell, applying log transformation and retaining only highly variable genes.

For the reconstruction algorithm, we scaled *L*_2_ of each cell to 1 to ensure that the circulant matrix has constant diagonal. For the gene inference algorithm, scaling *L*_2_ of each gene to 1 should be applied, as the score of each gene is relative to the rest of the genes. To estimate *α*, representing the correlation between neighbors according to the target topology, we search for the *α* value that best matches the spectrum of the given gene expression matrix. The results were improved by applying the algorithms after removing the theoretical covariance vector associated with the largest eigenvalue. For the cyclic case, the values of this eigenvector are constant.

### scPrisma general-case algorithm

1. Choose the desired topology (for example, periodic/linear). Calculate the theoretical covariance eigenvectors and eigenvalues.
2. Preprocess the data.
3. Reconstruct the signal by reordering the gene expression rows by solving Problem 2 (below) or by using prior knowledge.

Option 1—signal enhancement:

- Infer informative genes by solving Problem 3 (below) or by using prior knowledge and remove the rest of the genes.
- Enhance the desired signal by solving Problem 4 (below).

Option 2—signal filtering:

- Filter out the desired signal by solving Problem 5.

### Signal reconstruction

With a closed formula for the spectrum (2) and (3), we can estimate the pseudotime of the underlying cyclic trajectory. This can be done by estimating the rows reordering of the gene expression matrix that maximizes the projection over the theoretical spectrum. This problem can be formulated as a matrix permutation problem.

#### Problem 1

Matrix permutation problem for estimating a pseudotime that maximizes the projection over the theoretical spectrum:

$$\begin{array}{ll}&\arg \max E\,\,\,\, \mathop{\sum }\limits_{i=0}^{n-1}{\lambda }_{i}* {\overrightarrow{v}}_{i}^{\rm{T}}* (E* A)* {(E* A)}^{\rm {T}}* {\overrightarrow{v}}_{i}\\ &{\mathrm{s.t}}\, \, E\in P\end{array}$$

where *A* is the original gene expression matrix, ${\overrightarrow{v}}_{i}$ and *λ*_*i*_ are the theoretical eigenvectors and corresponding eigenvalues, respectively, and *P* is the set of permutation matrices. Under the assumption that *A* has a permutation *Ẽ* such that the spectrum of $({\tilde{\rm {E}}}*A)*({\tilde{\rm {E}}}*A)^{\rm{T}}$ matches the theoretical spectrum, the optimal solution is *E* = *Ẽ* (Supplementary Note A.15). Now, consider an identical formulation of the function we wish to maximize: $\mathop{\sum }\nolimits_{i = 0}^{n-1}{\lambda }_{i}* {{\left\Vert {(E* A)}^{\rm{T}}* {\overrightarrow{v}}_{i}\right\Vert }_{2}^{2}}$. This formula aims to maximize the product of each gene sorted by the permutation matrix and each theoretical eigenvector multiplied by its eigenvalue. These theoretical eigenvectors, as they are the eigenvectors of the theoretical covariance matrix, represent the variance along the theoretical topology. As a result, the permutation which maximizes this objective maximizes the variance along the theoretical topology.

Permutation problems are known to be NP-Hard^39^. We will follow previous studies in solving a convex relaxation of this problem, and instead of searching for a permutation matrix, we will search for a doubly stochastic matrix (the Birkhoff polytope)^39,40^:

#### Problem 2

Convex relaxation of Problem 1:

$$\begin{array}{ll}&\arg \max E\,\,\,\, \mathop{\sum }\limits_{i=0}^{n-1}{\lambda }_{i}* {\overrightarrow{v}}_{i}^{\rm{T}}* (E* A)* {(E* A)}^{\rm{T}}* {\overrightarrow{v}}_{i}\\ &{\mathrm{s.t}}\, \, \,{\overrightarrow{1}}^{\rm{T}}* E=\overrightarrow{1},\\ &E* \overrightarrow{1}=\overrightarrow{1}\end{array}$$

Here the objective function is quadratic and convex (Supplementary Note A.16). Despite the fact that maximizing it is not convex optimization, previous studies have shown that such problems can be efficiently resolved with stochastic gradient descent^41,42^. To project into the Birkhoff polytope, we used Bregmanian bi-stochastication algorithm^40^. Finally, for rounding the doubly stochastic matrix to a permutation matrix, we used a simple greedy algorithm. Specifically, the algorithm iterates over all rows, for each row rounds the maximum entry in each column that does not have 1 value yet to 1 and rounds to 0 the rest of the entries. The output of this algorithm is the permuted gene expression matrix: *A*_ordered_ = $E*A$.

### Genes inference

Once the reconstructed signal is obtained, either by solving Problem 2 or by prior knowledge, identification of informative genes that are related to the desired signal is possible. This can be achieved by filtering genes that do not maximize the projection over the theoretical spectrum. Because of convexity considerations, it would be easier to infer genes that are not related to the desired signal and then flip the results. Inference of the genes that are not related to the desired signal can be done by solving the following optimization problem:

#### Problem 3

Genes inference:

$$\begin{array}{ll}&\arg \min D\,\,\,\, \mathop{\sum }\limits_{i=0}^{n-1}{\lambda }_{i}* {\overrightarrow{v}}_{i}^{T}* ({A}_{{\mathrm{ordered}}}* D)* {({A}_{{\mathrm{ordered}}}* D)}^{T}* {\overrightarrow{v}}_{i}-{\gamma * \left\Vert D\right\Vert }_{1}\\ &{\mathrm{s.t}}\,\,\,D\,\,{\mathrm{is}}\,\,{\mathrm{diagonal}}\\ &0\le D\le I\end{array}$$

Because genes are represented by columns of *A*, each entry on the diagonal of *D* represents the influence of the respective gene on the spectrum. The number of filtered genes can be controlled by adding regularization. We can either increase the regularization coefficient, *γ*, to filter fewer genes or decrease it to filter more genes. The output of this algorithm is the gene expression matrix, after nullifying the genes that are not informative relative to the signal: *D*_1_ = *I* − *D*, *A*_gene-inferred_ = *A*_ordered_ $*$ *D*_1_.

### Filtering and enhancement

After inferring the set of informative genes (the genes related to the reconstructed signal), the next step is to remove any information that is not related to the signal, from the expression of those genes. This can be achieved by removing the diagonal constraint from Problem 3 and replacing the matrix product by the Hadamard product (element-wise product). For every entry in the expression matrix, *A*_*i*,*j*_, this formulation matches an optimization variable *F*_*i*,*j*_. Therefore, the enhanced gene expression matrix contains only expression profiles that maximize the projection over the theoretic spectrum.

#### Problem 4

Signal enhancement:

$$\begin{array}{ll}&\arg \max F\,\,\,\, \mathop{\sum }\limits_{i=0}^{n-1}{\lambda }_{i}* {\overrightarrow{v}}_{i}^{\rm{T}}* ({A}_{{\mathrm{genes-inferred}}}\odot F)* {({A}_{{\mathrm{genes-inferred}}}\odot F)}^{\rm{T}}* {\overrightarrow{v}}_{i}-\gamma * {\left\Vert F\right\Vert }_{1}\\ &{\mathrm{s.t}}\,\,\,0\le {F}_{i,j}\le 1\,\,\,\forall \,i,j\end{array}$$

Because this problem is not a convex optimization problem, it can be solved by using stochastic gradient ascent (adding noise at each iteration^43^). The output of this algorithm is the gene expression matrix, after eliminating information that is unrelated to the signal of interest: *A*_enhanced_ = *A*_genes-inferred_ ⊙ *F*.

Another option is similar to that described for Problem 3, which is to transform this problem into a minimization problem for filtering the reconstructed signal, thus turning it into a convex optimization problem (Supplementary Note A.16). Formulating this problem as a minimization problem eliminates the variance along the theoretical topology.

#### Problem 5

Signal filtering:

$$\begin{array}{ll}&\arg \min F\,\,\,\,\mathop{\sum }\limits_{i=0}^{n-1}{\lambda }_{i}* {\overrightarrow{v}}_{i}^{\rm{T}}* ({A}_{{\mathrm{ordered}}}\odot F)* {({A}_{{\mathrm{ordered}}}\odot F)}^{\rm{T}}* {\overrightarrow{v}}_{i}-\gamma * {\left\Vert F\right\Vert }_{1}\\ &{\mathrm{s.t}}\,\,\,0\le {F}_{i,j}\le 1\,\,\,\forall \,i,j\end{array}$$

The output of this algorithm is the gene expression matrix, after eliminating the information that is related to the signal of interest: *A*_filtered_ = *A*_ordered_ ⊙ *F*.

### Reporting summary

Further information on research design is available in the Nature Portfolio Reporting Summary linked to this article.

## Online content

Any methods, additional references, Nature Portfolio reporting summaries, source data, extended data, supplementary information, acknowledgements, peer review information; details of author contributions and competing interests and statements of data and code availability are available at 10.1038/s41587-023-01663-5.

### Supplementary information

Supplementary Information Supplementary Notes A.1–A.16, Figs. 1–10, and References.

Reporting Summary

Supplementary Table 1 List of gene regulatory interactions related to the circadian rhythm signal inferred by GRNBoost2 after cyclic enhancement by scPrisma.

Supplementary Table 2 List of cell–cell interactions related to the circadian rhythm signal inferred by CellPhoneDB after cyclic enhancement by scPrisma.

## Data Availability

The scRNA-seq datasets used for this study were acquired from the Gene Expression Omnibus (GEO) database with the following accession numbers: HeLaS3 (GSM4224315), liver (GSE145197), *Chlamydomonas* (GSE157580), SCN (GSE117295) and hESC (GSE64016). Slide-seqV2 dataset of the mice hippocampus was generated from ref. ^32^ and downloaded using Squidpy^44^. Pancreas datasets were generated from refs. ^26–29^ and downloaded using Scanpy^45^.

## Code availability

The code for scPrisma is publicly available at https://github.com/nitzanlab/scPrisma/
