# Supplementary material for: scPrisma infers, filters and enhances topological signals in single-cell data using spectral template matching
Source: Nat Biotechnol. 2023 Feb 27;41(11):1645–54. doi: 10.1038/s41587-023-01663-5 (PMC10635821; doi:10.1038/s41587-023-01663-5)
Supplement: Supplementary file 1 — Supplementary Notes A.1–A.16, Figs. 1–10, and References. [file 41587_2023_1663_MOESM1_ESM.pdf]

nature biotechnology

Article

https://doi.org/10.1038/s41587-023-01663-5

scPrisma infers, filters and enhances topological signals in single-cell data using spectral template matching

In the format provided by the authors and unedited

scPrisma infers, filters, and enhances topological signals in single-cell data using spectral template matching

Jonathan Karin[1] Yonathan Bornfeld[1] Mor Nitzan[123*]

[1]School of Computer Science and Engineering, The Hebrew University of Jerusalem
[2]Racah Institute of Physics, The Hebrew University of Jerusalem, Jerusalem, Israel
[3]Faculty of Medicine, The Hebrew University of Jerusalem, Jerusalem, Israel
*Correspondence: mor.nitzan@mail.huji.ac.il

A scPrisma- Supplementary Notes

A.1 scPrisma identifies and filters periodic signals

As a starting point, we assess the performance of scPrisma on simulated data and show its ability to reconstruct underlying cyclic signals, infer cyclic genes, as well as filter or enhance cyclic signals. We first simulated noiseless gene expression matrices encoding a cyclic signal (Supplementary A.2). scPrisma successfully captures the cyclic signal (Extended Data Figure 1A), and the reconstruction is robust to noise, as the quality of reconstruction gradually decreases with decreasing SNR (signal to noise ratio, $\frac{P_{signal}}{P_{noise}}$, where we use Frobenius norm as power), and deteriorates only at high noise levels ($SNR < 0.25$) (Extended Data Figure 1B; Supplementary A.3). Using scPrisma, we can also correctly classify the subset of cyclic genes and distinguish them from genes expressing Gaussian noise (Supplementary A.3). Further, in a more challenging setting, when entangling the cyclic signal with a hierarchical signal (that can capture lineage relationships within the data, following the model suggested in [23]; Supplementary A.3), our approach can still identify the subset of cyclic genes, in a way that is robust to increasing noise (Extended Data Figure 1C).

Next, to demonstrate the capabilities of our approach for either filtering or enhancing underlying cyclic signals in the data, we simulated noisy gene expression matrices with embedded cyclic as well as linear signals (Methods). scPrisma can both enhance, as well as filter the cyclic signal, and thus, either weaken or retain and effectively enhance the linear signal in the data, respectively. For this task we benchmarked scPrisma relative to Cyclum[17], which supports the inference and filtering of circular components in scRNA-seq data. scPrisma robustly outperforms Cyclum in both enhancement of the cyclic signal (mean MSE between the original gene expression matrix and the cyclic signal reduces by 44% using scPrisma, compared to nearly 0% using Cyclum, and 38% using ground-truth reconstruction, Extended Data Figure 1E), as well as filtering of the cyclic signal, thus revealing the underlying linear signal in the data (mean MSE between the original gene expression matrix and the linear signal reduces by 33% using scPrisma, compared to 27% using Cyclum, and 40% using ground-truth reconstruction, Extended Data Figure 1E). Furthermore, when simulating a cyclic signal with varying amount of Gaussian noise, we found a substantial improvement in recovering the underlying cyclic signal following its spectral enhancement (Supplementary Figure 1C). Finally, when simulating single-cell data combining linear and hierarchical signals, and using scPrisma's linear spectral analysis (Methods), we find that the inference of genes associated with the linear process is robust and degrades only at high noise levels ($SNR < 0.25$) (Supplementary Figure 1E,F).

A.2 Cyclic models

In the first model - the progression model (which is described in the main text, and illustrated in Supplementary Figure 10), we start with a 'root' cell. Next, we duplicate the existing cell, select uniformly k genes, and switch their states. This process is repeated q times. Then, within the last generated cell, k genes whose state differs from the 'root' cell are randomly chosen, their state is switched and the cell is duplicated. The process is repeated until the gene expression of the newest cell is identical to that of the original root cell.

In the second model - the spatial model (illustrated in Supplementary Figure 10B), we receive as input the number of cells (n), the numbers of genes (p) and a window size ($0 < w < 1$). The simulation starts by arranging the n cells on a circle and initializing the gene expression of each one of them as 'OFF' for all genes. Then, for each gene we choose uniformly the center cell and switch the gene to 'ON' for the cells inside the window of size $w * n$ around the center cell.

The third model is similar to the second model, but instead of step functions of gene expression around each center cell, expression is estimated by a Gaussian distribution centered at the center cell.

A.3 Simulated data

For the reconstruction evaluation, we simulated a cyclic signal according to the spatial model (Supplementary A.2). The signal consists 100 cells and 500 genes with w=0.3. We did not shuffle the rows, so the ground truth order is the identity permutation (or any other shift, or shift of the reverse order). For 300 iterations we added Gaussian noise with varying variance, and used the reconstruction algorithm. Next, the permutation matrix was transcribed to permutation array. And finally, the maximum Spearman rank correlation was taken between the output permutation array and every possible shift and inverse shift of the identity permutation.

For the genes inference evaluation, cyclic signal of 256 cells and 250 genes was simulated according to the spatial model, And a lineage signal of 256 cells and 250 genes was simulated according to the model described by Nitzan & Brenner [23]. Again, for 300 iterations we added Gaussian noise with varying variance, and used the genes inference task. As a ground truth vector, cyclic genes were labeled as '1' and the lineage genes were labeled as '0'. The AUC-ROC of the diagonal of D matrix and the ground truth vector were calculated.

A.4 HeLa cells data

The HeLa cells data was generated by [29]. We used similar pre-processing as in [37], including normalization per cell, log transformation and selection of the top (7000) highly variable genes. Additionally, similarly to [29], we filtered out cells with low number of counts, until the mean counts reached 4,500 UMI counts per cell. In this section, as we evaluate the gene inference task directly following the application of scPrisma, we did not perform gene inference before the spectral enhancement of the data. The polar plot (Figure 2B) consists of 5 different phases (G1.S, S, G2, G2.M, M.G1), where for each phase we used a list of corresponding marker genes[29]. For each phase, we created a vector that represents the probability distribution of each cell to be in each cell cycle phase. To calculate those vectors, we summed all the phase-related genes and then normalized the sum of each vector to 1. To evaluate the performance of the algorithms, we measured two terms for each vector: circular mean ($\bar{\alpha}$) and circular variance[14]. In angular statistics, circular mean is a measure that reflects the mean of a set of angles or similar cyclic quantities. Given a set of observed angles: $\alpha_1, ...\alpha_n$, the circular mean is $\bar{\alpha} = atan2(\frac{1}{n}\sum_{i=0}^{n-1}\sin\alpha_i, \frac{1}{n}\sum_{i=0}^{n-1}\cos\alpha_i)$ ($atan2(x, y)$ returns a value θ between $-\pi \le \theta \le \pi$, where for some $r > 0$: $x = r\cos\theta, y = r\sin\theta$). In our case, given an ordering of the cells in a cycle (according to the reconstruction algorithm), we have n (the number of cells) samples that are distributed uniformly along the cycle, but the probability (the weight) of each one of them is different, and the total weights are summed to 1. So the circular mean would be: $\bar{\alpha} = atan2(\sum_{i=0}^{n-1} w_i * \sin(\frac{i}{n}2\pi), \sum_{i=0}^{n-1} w_i * \cos(\frac{i}{n}2\pi))$, where w_i is the i'th entry in the expression vector (related to the i'th cell). Circular variance measures the spread of dihedral angles. The circular variance ranges from 0 to 1, where lower values reflect clustering of the samples around the circular mean. The circular variance is defined as $Var = 1 - \bar{R}/n$, where $\bar{R}^2 = (\sum_{i=0}^{n-1}\sin\alpha_i)^2 + (\sum_{i=0}^{n-1}\cos\alpha_i)^2$. In our case, since the distribution is not uniform we will calculate: $Var = 1 - \bar{R}$, where $\bar{R}^2 = (\sum_{i=0}^{n-1} w_i * \sin\alpha_i)^2 + (\sum_{i=0}^{n-1} w_i * \cos\alpha_i)^2$.

To evaluate the informative genes inference task, we used sets of 200 genes, composed of (1) random subsets of 100 genes from the list above, and (2) 100 genes that do not appear on the list (50 iterations).

Last, we showed that scPrisma does not generally overfit non-cyclic genes to the circular topology (Supplementary Figure 2C).

A.5 Liver data

The mouse Liver data was generated by [6], and the lobule layers were reconstructed based on [11]. We used standard pre-processing, consisting of normalization per cell, log transformation and selection of

the top 7,000 highly variable genes. We subsampled the data for even temporal distribution by selecting 1,000 cells from each timepoint. To label the cells according to their position along the lobule axis, we used the algorithm in [6]. The output of this algorithm is a probabilistic embedding, where each cell was eventually labeled by the layer associated with the highest probability. In addition to the enhancement analysis which is described in the main text (which we will term here 1-step enhancement), we applied cyclic (linear) enhancement following linear (cyclic) filtering (which we will term 2-step enhancement). We found that the 2-step enhancement does not improve the results obtained by the 1-step enhancement: K=8 KMeans for linear enhancement following/without cyclic filtering is 0.38/0.5, respectively (compared to 0.11 for raw data), and K=4 KMeans for cyclic enhancement following/without linear filtering is 0.95/0.96, respectively (compared to 0.11 for raw data). Last, we compared the performance of scPrisma for filtering the cyclic signal with those of Seurat and Cyclum. For Seurat [4], we used the circadian clock genes list provided by [6]. Filtering the circadian rhythm signal is a challenging task since it is weaker here than the spatial zonation signal [6], and therefore cannot be easily reconstructed. While scPrisma successfully filtered the circadian rhythm (Figure 3), the alternative two methods, Cyclum and Seurat, were ineffective in filtering out the circadian rhythm signal; ARI of K=4 KMeans: raw data 0.11, scPrisma filtering 0.013, Cyclum filtering 0.10 and Seurat filtering 0.10.

A.6 Chlamydomonas data

The Chlamydomonas data was generated by [20]. We used standard pre-processing, consisting of normalization per cell, log transformation, and selection of the top 7,000 highly variable genes. We subsampled the data for even condition-based distribution by selecting 3,000 cells from each condition.

To verify that our reconstruction corresponds to the diurnal cycle, we used a list of marker genes that was obtained from bulk RNA-seq[32], where the peak times of genes that are influenced by the diurnal cycle are annotated. We divided the 24-hours diurnal cycle to 6 equally-spaced segments (0-3.5,4-7.5,8-11.5,12-15.5,16-19.5,20-23.5). Then, similarly to the HeLa cells analysis (Supplementary A.4), the genes were summed and normalized by dividing all values by the maximum value.

A.7 SCN data

The SCN data was generated by [19]. We used a dataset of 12 samples taken at equal intervals along 48 hours. We treated samples separated by a 24-hour interval as sampled at the same circadian time. We clustered the cells according to cell types by using Louavin algorithm, where the mapping between clusters to cell types was done using established cell type marker genes[19]. We used *Agt* for astrocytes, *Itm2a* for endothelial, *Tmem212* for ependymal, *Hexb* for microglia, *Celf4* for neurons, *Pdgfra* for NG2, *Plp1* for oligodendrocytes, and *Col23a1* for tanyceytes. The expression of each marker gene in each cluster is shown in Supplementary Figure 6. In addition, our analysis of the neural population concentrated on the SCN neurons (which can be identified by the marker genes: *Vip, Avp, Nms, and Cck*), as they exhibit clear temporal peaks of the core clock genes (mean circular variance 0.033), in contrast to nearly flat expression of these genes in the remaining neurons (mean circular variance 0.154).

It can be observed (Figure 5A) that cellular density varies across the different time points, which seems to reflect underlying biological variability as time points that were previously identified as peaks of the rhytmic process[19] exhibit higher cellular concentration. For example, for ependymal cells, the timepoints CT06, CT10 and CT14 exhibit high cellular concentrations (Figure 5A), while being reported as the peaks of the rhythmic process using gene ontology[19], consistent with rhythmic genes mainly expressed at these timepoints, such as *Tef* which is known to play a role in circadian regulation[36], and is mainly expressed around CT10 and CT14 (Figure 5B).

We recovered the neuronal subtypes by clustering using the Leiden algorithm. According to [19], there are four main SCN neuron subtypes: 'N0' (marker genes: *Avp/Nms*), 'N2' (marker genes: *Avp, Cck*; can be divided into two subgroups), 'N6' (marker genes: *Vip, Nms, C1ql3*), and 'N9' (marker genes: *Vip, Grp*). Focusing on N0 and N2, When running a naive clustering algorithm (Leiden) over the cells that were classified as neurons, we get 13 clusters. The marker genes of N0 and N2 are expressed in 4 of these clusters: 1,3,4 and 12 (where cluster 12 contains only 293/14766 cells). We conclude that clusters 1,3 and 4 contain a mixture of N0 and N2 cells, since all three marker genes (*Avp/Nms/Cck*) are expressed in all three clusters. Moreover, the majority of cells in clusters 1 and 3 (79% and 66%, respectively) are sampled in the timepoints CT =14/18/22, while the majority of cells in cluster 4 (93%) are sampled in the timepoints CT = 02/06/10. Only after cyclic filtering, can cluster 0 be uniquely classified as N0 (according to *Nms* expression), while cluster 2 can be classified as N2 (according to *Cck*

expression) (Figure 5E).

We evaluated the prediction of regulatory interactions associated with the circadian rhythm, using the GRNBoost2 algorithm, relative to a baseline circadian network [21]. For each cell type separately, we summed the scores of all regulatory interactions in the circadian network.

Finally, we used scPrisma to infer hidden cell-cell interactions related to the circadian rhythm. We compared cell-cell communication patterns, based on inferred ligand-receptor interactions using CellPhoneDB database and method [8], between different cell types at corresponding time points. Similarly to the regulatory network inference, we searched for interactions that were substantially enhanced following scPrisma's cyclic analysis (Methods) (The full list of predicted cell-cell interactions can be found in Supplementary Table 2). Such enhanced interactions are expected to be enriched for those associated with the circadian rhythm, or rhythmic processes more generally. We specifically searched for interactions whose score increased at least two-fold following scPrisma's enhancement (Supplementary A.7). An example of such enhanced inferred interaction is between *Avp* in astrocytes and *Plau* in microglia, both known to be associated with the circadian rhythm [25, 24], whose mean score in CT18 was increased following enhancement from 0.501 to 1.011 (p-value=0.003 before enhancement, p-value < 0.001 after enhancement) (Methods, Figure 5I). More generally, we searched for interactions associated with rhythmic processes using the corresponding gene ontology categorization (rhythmic process, GO:0048511)[12], and termed cell-cell interactions mediated by a receptor (ligand) which is associated with a rhythmic process as receptor (ligand) -mediated rhythmic interactions. The percentage of inferred receptor-mediated rhythmic interactions which were substantially enhanced (at least 2-fold, as above) following cyclic enhancement increased to 16.32%, relative to 11.61% before enhancement. Similarly, the percentage of ligand-mediated rhythmic interactions increased to 14.28% relative to 9.68% before enhancement. In summary, the results for predicted gene regulatory interactions and cell-cell interactions following cyclic enhancement by scPrisma (detailed in Supplementary Tables 1,2) are expected to reveal and strengthen previously overshadowed interactions related to cyclic processes such as the circadian rhythm, which is supported by the results over the subset of known core clock regulatory interactions, as well as enrichment of rhythmic genes among ligands and receptors of predicted cell-cell interactions.

A.8 Comparisons

To compare the filtering and the enhancement of the simulated data by scPrisma relative to Cyclum[17], we used the defualt parameters of Cyclum (with the same pre-processing as scPrisma), except for the dimensionality of the embedding layer. To find the optimal embedding layer, we ran it with eleven different values for dimensions $\{1, 2, ..11\}$ as suggested in the original paper, and chose the best result.
To compare the filtering of the diurnal cycle, we used Cyclum as described for the simulated data. For comparison with Seurat [4] and ccRemover [2]), we used the marker genes list of the diurnal cycle that was provided by[32]. For ccRemover we used the whole list. For Seurat we sorted the genes according to their peak phase, divided the genes list to two ranked subsets randomly (over 50 iterations), and chose the best result.

A.9 Regularization parameter

In our method, the regularization parameter, γ, is the only significant free parameter, and incorrect selection of this parameter can result in non-optimal filtering. We recommend using $\gamma = 0$ as the starting point for all algorithms. If the enhancement algorithm does not filter out enough information, it is recommended to increase the regularization parameter incrementally by $1/n$, where n is the number of cells. For the filtering algorithm, if the algorithm filters out too much information, it is recommended to increase the regularization parameter incrementally by $1/n$. For the gene inference task, we recommend increasing the regularization parameter incrementally by 0.1 until a suitable number of genes are retained. Another way to tune the regularization parameter of the gene inference task is based on prior knowledge of genes that are expected to be filtered out (e.g the cell type markers in Section 2.5) and genes which are expected to be retained (e.g the core clock genes in Section 2.5). This can be done, for example, by gradually increasing the regularization parameter (as suggested above) until the point where all genes which are expected to be unrelated to the target process according to prior knowledge are filtered out.

A.10 Integrating prior knowledge for the reconstruction problem

Since the reconstruction task is challenging, integrating any available prior knowledge can be helpful, such as restricting the optimization parameter (E) to a convex subset of the doubly stochastic matrices set.

For example, we simulated a cyclic signal with varying amount of noise, and applied the reconstruction algorithm both without prior knowledge (de novo) and with prior knowledge related to low-resolution pseudotime ordering (specifically, division of the cells to three consecutive temporal bins). We find that integration of prior knowledge in this case renders the algorithm robust to high noise levels ($SNR < 0.1$), as opposed to the de novo version (Supplementary Fig 1E).

Another possible type of prior knowledge that can be integrated is related to gene selection, where we can apply the reconstruction algorithm only over a subset of genes that are known to be related to the desired signal. For example, in many scRNA-seq datasets, cell type is a major source of variation reflected in the covariance matrix, and therefore, better results are generally expected if the reconstruction algorithm of the cell cycle is applied after selecting for genes that are known to be related to this process.

A.11 Estimating α

As a prior step for constructing the analytical cell-cell covariance matrix, we need to estimate the free parameter α ($\alpha(1)$). According to the theoretical model, based on the model described in [23, 27], the covariance between two cells whose distance over the topology is m is modeled as $\alpha(m) = E[X(m)X(0)] = \exp(-2mk/p)$, where p is the number of features (genes in our case) and k is the number of changes between neighboring cells. In real datasets, k is expected to decrease with the density of cells over the manifold, and in addition, is a function of the sequencing depth and number of sampled cells.
For scPrisma, α is estimated by optimizing the fit to the eigenvalues spectrum of the data. This can be done by solving the following optimization problem:

Problem 6. *Estimating α*
$\arg\min \alpha \quad \left\| \sum_{i=0}^{q-1} \lambda_i - \hat{\lambda}_i(\alpha) \right\|$
$s.t \quad 0 \leq \alpha \leq 1,$

where λ_i is the i'th largest eigenvalue of the numerical covariance matrix of the dataset and $\hat{\lambda}_i(\alpha)$ is the i'th largest eigenvalue of the theoretical covariance matrix. An alternative is to use a fixed k (e.g. $k = 2$ as in [23]).

A.12 Evaluating the performance of the reconstruction algorithm

We discuss several approaches to assess whether the output of the reconstruction algorithm is meaningful and whether the target topology could be detected in the data.

First, as validation, if topologically informative genes (or, signal-specific marker genes) are available, one could assess whether their expression is topologically smooth following reconstruction, as expected (as we demonstrated, for example, in Sections 2.2 and 2.4).

Second, we offer a direct measure for the convergence of the reconstruction algorithm, based on the projection proportion score. The projection over the theoretical spectrum (which lies at the core of scPrisma) is defined as $\sum_{i=0}^{n-1} \lambda_i * \vec{v}_i^T * A * A^T * \vec{v}_i$, where A is the gene expression matrix, and the theoretic eigenvalues and eigenvectors are $\lambda_1, \lambda_2 ... \lambda_n$, and $\vec{v}_1, \vec{v}_2, .. \vec{v}_n$, respectively. As is exemplified in Supplementary Figure 9A, the value of this projection is indeed higher for data containing the target topology (such as cyclic Gaussian-based gene expression matrices; Third model, Section A.2), relative to data that does not contain it (such as gene expression matrices of random Gaussian noise). The reconstruction algorithm searches for the cellular permutations that maximize the value of this projection, and thus, if no permutation corresponds to the target topology, the projection score will not substantially increase following the reconstruction. Therefore, we use the proportion between the projection value before and after reconstruction as a measure corresponding to a meaningful topological convergence of the reconstruction algorithm. More formally, we define the projection proportion score as:
$\sum_{i=0}^{n-1} \lambda_i * \vec{v}_i^T * (A) * (A)^T * \vec{v}_i / \sum_{i=0}^{n-1} \lambda_i * \vec{v}_i^T * (E * A) * (E * A)^T * \vec{v}_i,$
where E is the inferred permutation matrix. As an example, we added Gaussian noise with varying variance over the HeLa cells dataset[29] (Section 2.2). Increased noise variance leads to higher projection proportion score and lower AUC score, as expected (Supplementary Figure 9B). For the SCN dataset (Section 2.5), the projection proportion score corresponds to the ability to reveal the circadian rhythm signal across different cell types, as expected (Supplementary Figure 9C). Specifically, cell types that exhibited a clear cyclic signal following cyclic enhancement by scPrisma (Figure 5, Supplementary Figure 7) exhibit lower projection proportion scores than the three cell types that did not expose a clear cyclic signal following cyclic enhancement (NG2, microglia, and tancytes; those which exhibit the lowest

fraction of rhythmic genes expression out of total expressed genes [19]). The projection proportion score for other datasets analyzed in the manuscript, for which a cyclic signal (corresponding to a meaningful cyclic biological process) could be recovered, is close to 0; Specifically, the projection proportion score is 0.0723 for the HeLa dataset (Section 2.3), 0.0093 for the liver dataset (Section 2.3), and for the Chalamydomonas data (Section 2.4) the projection proportion score is 0.0133 and 0.011, for the FE- and FE+ conditions, respectively.

An additional approach to assess the existence of the target topology in the data, following the reconstruction algorithm, is to apply scPrisma's enhancement workflow (including gene inference) and using the L2 entry-wise norm of the final gene expression matrix as a measure corresponding to the role of enhancement in this case. We demonstrate this in a controlled case over simulated data. Specifically, we generated a 100X100 matrix of Gaussian noise (mean=0, variance=0.5, the expression matrix was clipped to be positive), and added a variable number of cyclic genes from a 100X100 matrix generated according to our cyclic model (Methods). Then, we normalized the matrix L2 entry-wise norm to 1 and applied the full scPrisma enhancement workflow. As expected, as the signal strength (fraction of cyclic genes compared to noisy genes) increases, the gene inference algorithm filters out decreasing fractions of the data, and the gene expression norm following enhancement increases (with a form that depends on the regularization coefficient) (Supplementary Figure 9).

A.13 Topological priors on clusters, multiple cyclic processes, and spatial data

A.13.1 Clustered structure

To enhance or filter the distinction between different cellular clusters, we applied scPrisma with a block-structure covariance matrix A. Specifically, A is defined as follows: $A_{i,j} = 1$ if i and j are in the same cluster, and $A_{i,j} = 0$ otherwise. The spectrum of A can be analyzed analytically. For example, given two clusters (c_0 and c_1), the spectrum of A contains two eigenvalues different from 0, $\lambda_0 = |c_0|$ and $\lambda_1 = |c_1|$, with corresponding eigenvectors v_0 and v_1, where for $j = \{0, 1\}$, $v_{j_i} = 1/\sqrt{|c_j|}$, for $i \in c_j$ and 0 otherwise.

For the batch correction of multiple single-cell datasets of human pancreas [30, 1, 35, 22], we followed benchmarking that was done by [18], calculating the Silhouette score (ASW) of 50 PC's between the different batches after applying batch correction by multiple state-of-the-art computational methods (lower score corresponds to better filtering of differences between batches). We find that scPrimsa (-0.0919) is comparable to scGen [18] (-0.0917), and outperforms Harmony [15] (-0.0417), scanorama [13] (-0.0074), MultiCCA [4] (-0.0009), and MNN [10] (0.0087).

A.13.2 Multiple cyclic processes

Enhancing multiple, phase-shifted cyclic processes in the SCN cellular population [19] is challenging and cannot be resolved by shifting the temporal expression of certain cell types relative to the neurons, since there are different subsets of genes that are either synchronized (such as protein folding genes) or unsynchronized (such as core clock genes) along the circadian rhythm. Therefore, to enhance the cyclic signals of two different cell types simultaneously, we design a block circulant matrix as a covariance matrix for scPrisma. The block circulant matrix contains four blocks: the upper left (lower right) block is a circulant matrix of the size of the number of cells of cell type 1 (2), the upper right and lower left blocks are uniform matrices of value that is equal to the mean value of the circulant blocks.

To infer multiple processes encoded by the same cells, we used a hESC (human embryonic stem cells) single-cell dataset which contains 213 H1 single cells [16]. We showed that scPrisma can be used to iteratively reveal cyclic processes in this context (de novo, without knowledge of process-related marker genes). First, we applied scPrisma's circular enhancement pipeline de novo (as explained in the Methods section). We found that the recovered cycle corresponds to the cell cycle, based on the reduced circular variance and increased Moran's I score of cell cycle marker genes chosen in the original study[16] (Tables 1, 2, Supplementary Figure 8). Next, we used scPrisma's filtering algorithm (on the first-round reconstructed data) to filter the first cyclic signal. Taking the filtered data as input, we again applied scPrisma's circular enhancement pipeline de novo. We found that the recovered cyclic signal corresponds to an oscillatory pattern related to the experimental setup (chip capture site and output well positions on the Fluidigm C1 chip used for the experiment), based on the reduced circular variance and increased Moran's I score of the signal-related marker genes recovered in the original study[16] (Tables

1, 2, Supplementary Figure 8). The encoding of both oscillatory processes by the hESC population is consistent with the findings of [16].

Stage	Cell cycle				Capture site			
	NUSAP1	*KPNA2*	*CCNB1*	*TPX2*	*RPL13*	*MIF*	*PFN1*	*COX5A*
Raw data	0.952	0.985	0.975	0.955	0.992	0.989	0.975	0.963
First reconstruction	0.826	0.902	0.876	0.789	0.990	0.974	0.953	0.958
Enhancement of the first signal	0.495	0.345	0.388	0.570	Filtered	Filtered	Filtered	Filtered
Second reconstruction	0.983	0.989	0.988	0.979	0.897	0.884	0.898	0.849
Enhancement of the second signal	Filtered	Filtered	Filtered	Filtered	0.406	0.421	0.512	0.801

Table 1: Circular variance of marker genes following the usage of scPrisma over the hESC dataset.

Stage	Cell cycle				Capture site			
	NUSAP1	*KPNA2*	*CCNB1*	*TPX2*	*RPL13*	*MIF*	*PFN1*	*COX5A*
Raw data	0.005	0.003	0.004	0.003	0.001	0.001	0.002	0.002
First reconstruction	0.065	0.108	0.087	0.069	0.001	0.003	0.006	0.002
Enhancement of the first signal	0.170	0.184	0.177	0.139	Filtered	Filtered	Filtered	Filtered
Second reconstruction	0.001	0.005	0.002	0.001	0.067	0.064	0.037	0.033
Enhancement of the second signal	Filtered	Filtered	Filtered	Filtered	0.173	0.178	0.156	0.053

Table 2: Moran's I score of marker genes following the usage of scPrisma over the hESC dataset.

A.13.3 2D spatial tissue organization

To infer and manipulate the spatial signal from spatially-informed data, we designed a spatial covariance matrix to be used by scPrisma as follows: We first computed the KNN graph of the cells based on their spatial coordinates (here we used K=20). Next, based on the KNN graph, we computed the shortest path matrix, and transformed it to an affinity matrix using a heat kernel ($C = \exp(-D)$, where D is the shortest path matrix), which is taken as a proxy for the covariance matrix for scPrisma. We used the enhancement workflow over Slide-seqV2 dataset of the mouse hippocampus [31] using the described spatial covariance matrix, to first infer spatially-informative genes using the gene inference algorithm, and then enhance spatially-related expression patterns. The spatial enhancement can be more useful than simple smoothing algorithms, as it does not overfit to the topology in a way that prevents downstream analysis (e.g. does not infer non-existing expression values).

In addition, we demonstrate scPrisma's ability to manipulate the spatial signal via its manipulation of gene expression of spatially proximate cell types. We focused on three pairs of cell types which are spatially proximate according to the highest neighborhood enrichment score [26]: Oligodendrocytes & Polydendrocytes, Endothelial_Tip & Ependymal, Endothelial_Tip & Endothelial_Stalk. Calinski and Harabasz scores [5] were used to validate that those pairs of cell types drift apart in the gene expression space following spatial filtering, and become closer following spatial enhancement. Over the raw data the Calinski and Harabasz scores between those cluster pairs are 164.39, 20.10 and 25.51, respectively. After filtering out the spatial signal the scores decrease, as expected, to 33.91, 11.83 and 6.05, respectively,

while after extracting the spatial component by spatial enhancement the scores increase to 590.13, 140.94 and 34.57, respectively.

For benchmarking, we filtered the data using Seurat's filtering feature [28], by taking as reference genes the top spatially variable genes according to Moran's I score. While the Calinski and Harabasz scores between those cluster pairs decrease following filtering by Seurat, it does not decrease to the same extent as enabled by scPrisma (Table A.13.3).

Method / Calinski and Harabasz score for cell types pairs	Raw data	scPrisma	Seurat (top 50 genes)	Seurat (top 100 genes)	Seurat (top 150 genes)	Seurat (top 500 genes)
Oligodendrocytes & Polydendrocytes	164.39	33.91	44.49	47.85	72.77	158.72
Endothelial_Tip & Ependymal	20.10	11.83	18.51	19.68	19.10	20.10
Endothelial_Tip & Endothelial_Stalk	25.11	6.05	26.13	26.01	25.44	25.51

Table 3: Comparison of the performance of filtering out the spatial signal of Slide-seqV2 dataset of the mouse hippocampus [31]. The Calinski and Harabasz scores were calculated before and after spatial filtering by scPrimsa and Seurat [28] for cell type pairs that are spatially proximate according to the highest neighborhood enrichment score.

Last, we analyzed the biological processes enriched in the data following scPrisma manipulation. Specifically, we analyzed the gene ontology enrichment (using GOrilla [7]) based on a list of 500 HVGs (extracted by Seurat V3 [33]) before and after spectral filtering. Before spatial filtering, the statistically significant GO terms are GO:0005179 (hormone activity p-value=3.07E-5) and GO:0048018 (receptor ligand activity p-value=8.05E-4) which are driven by spatially variable genes (*Ttr, Sst, Npy*; Moran's I score = 0.708/0.356/0.274, respectively). However, after filtering these terms diminish and the only statistically significant term is GO:0048029 (monosaccharide binding p-value=4.38E-4) which is driven by non spatially variable genes (*Clec4n*, Moran's I score = -0.0002, and *Siglec5*, which was not sampled in this experiment).

A.14 Runtime analysis and memory consumption

The algorithms included in scPrisma are based on simple matrix multiplication, and therefore can be scaled to large datasets. The algorithm with the largest computational complexity (and memory consumption) is the reconstruction algorithm due to two main reasons: first, it learns a permutation matrix of size of the number of cells squared, which can be memory-consuming. The second reason is the projection over the Birkhoff polytope (doubly stochastic matrices set). As mentioned in the manuscript (Methods), we used the Bregmanian Bi-Stochastication algorithm [34] which includes only matrix multiplications and therefore can be boosted using GPU. While challenging, it is not necessary for scPrisma, since the reconstruction can be provided as input, as in Sections 2.3 and 2.5. Overall, the scaling bottleneck of scPrisma is the hardware that supports matrix multiplication and especially the memory availability. For runtime and memory consumption analysis, we simulated a cyclic signal (Section A.2) of 2000 genes, with varying number of cells, and added Gaussian noise. We ran the simulation using a single GPU NVIDIA RTX A5000. The filtering/enhancement algorithms and the genes inference algorithm can easily scale to tens of thousands of cells, while scaling the reconstruction algorithm to large datasets is more challenging (Extended Data Figure 7).

A.15 Proof of correctness

Theorem 1. *Let* $A * A^T$ *(where* $A \in R^{n\times m}$*) be a circulant matrix with eigenvalues* $\lambda_1, \lambda_2 ... \lambda_n$*, and eigenvectors* $\vec{v}_1, \vec{v}_2, .. \vec{v}_n$*, and assuming that we have* $\tilde{A}$ *which is a rows permuted version of* A*, then* $E * \tilde{A} * (E * \tilde{A})^T$ *is circulant, where* E *is the solution of Problem 1.*

Proof. It is sufficient to show that given two permutation matrices E and $\tilde{E}$, if $E * \tilde{A} * (E * \tilde{A})^T$ is circulant with the eigenvalues $\lambda_1, \lambda_2 ... \lambda_n$, and eigenvectors $\vec{v}_1, \vec{v}_2, .. \vec{v}_n$, then:
$\sum_{i=0}^{n-1} \lambda_i * \vec{v}_i^T * (\tilde{E} * \tilde{A}) * (\tilde{E} * \tilde{A})^T * \vec{v}_i \leq \sum_{i=0}^{n-1} \lambda_i * \vec{v}_i^T * (E * \tilde{A}) * (E * \tilde{A})^T * \vec{v}_i$

Assuming that the eigenvectors of $(\tilde{E} * \tilde{A}) * (\tilde{E} * \tilde{A})^T$ are $\vec{u}_1, \vec{u}_2, ..\vec{u}_n$, and since eigenvalues are insensitive to permutations, the eigenvalues are $\lambda_1, \lambda_2 ... \lambda_n$.
Left side:
$\sum_{i=0}^{n-1} \lambda_i * \vec{v}_i^T * (E * \tilde{A}) * (E * \tilde{A})^T * \vec{v}_i \stackrel{1}{=} \sum_{i=0}^{n-1} \lambda_i * \vec{v}_i^T * (\sum_{k=0}^{n-1} \lambda_k * \vec{v}_k * \vec{v}_k^T) * \vec{v}_i$
$\sum_{i=0}^{n-1} \lambda_i * \vec{v}_i^T * (\sum_{k=0}^{n-1} \lambda_k * \vec{v}_k * \vec{v}_k^T) * \vec{v}_i \stackrel{2}{=} \sum_{i=0}^{n-1} \lambda_i^2$

Right side:
$\sum_{i=0}^{n-1} \lambda_i * \vec{v}_i^T * (\tilde{E} * \tilde{A}) * (\tilde{E} * \tilde{A})^T * \vec{v}_i = \sum_{i=0}^{n-1} \lambda_i * \vec{v}_i^T * (\sum_{k=0}^{n-1} \lambda_k * \vec{u}_k * \vec{u}_k^T) * \vec{v}_i$
$\sum_{i=0}^{n-1} \lambda_i * \vec{v}_i^T * (\sum_{k=0}^{n-1} \lambda_k * \vec{u}_k * \vec{u}_k^T) * \vec{v}_i = \sum_{i=0}^{n-1} (\sum_{k=0}^{n-1} \lambda_i * \lambda_k * \langle \vec{u}_k, \vec{v}_i \rangle^2)$
Therefore we need to show that : $\sum_{i=0}^{n-1} (\sum_{k=0}^{n-1} \lambda_i * \lambda_k * \langle \vec{u}_k, \vec{v}_i \rangle^2) \leq \sum_{i=0}^{n-1} \lambda_i^2$.
Ky Fan [9] (LEMMA 1A) proved a similar lemma:
Let $a_1 \geq a_2 \geq ....a_n \geq 0$, $b_1 \geq b_2 \geq ....b_n \geq 0$. If p_{ij} are n^2 non-negative numbers such that: $\sum_{i=1}^{n} p_{ij} \leq 1 \; \forall j \; \wedge \; \sum_{j=1}^{n} p_{ij} \leq 1 \; \forall i$,
then:
$\sum_{i=1}^{n} \sum_{j=1}^{n} p_{ij} * a_i * b_j \leq \sum_{i=1}^{n} a_i * b_i$.
In our case, we can use the lemma since from Bessel's inequality: $(\sum_{k=0}^{n-1} \langle \vec{u}_k, \vec{v}_i \rangle^2) \leq 1, \; \forall k \wedge (\sum_{i=0}^{n-1} \langle \vec{u}_k, \vec{v}_i \rangle^2) \leq 1, \; \forall i)$, and all the eigenvalues are non-negative since the covariance matrix is a PSD matrix. Therefore, we can use the lemma with: $a_i = \lambda_i, b_j = \lambda_j \forall i, j$.

□

A.16 Proof of convexity

To prove the convexity of the objectives of the optimization problems (Methods), we will use the following theorem:

Theorem 2. *$f \colon R \to R^n$ is convex if and only if $g \colon R \to R$ is convex. where $g(t) = f(x+tv)$,* $\quad$ **dom** $g = \{t | x \; + tv \in$ **dom** $f\}$*[3]*

.

Claim 2.1. *The objective function of the convex relaxation of the reconstruction problem (Methods 9.5) is convex.*
$f(E) = \sum_{i=0}^{n-1} \lambda_i * \vec{v}_i^T * (E * A) * (E * A)^T * \vec{v}_i$
$s.t \quad \vec{1}^T * E = \vec{1},$
$E * \vec{1} = \vec{1}$

Proof. The set iself is convex (linear equalities). Let us define $g(t) = f(E + tU)$, where $E + tU \in \mathbf{dom} f$, and calculate the first and second derivatives:
$g'(t) = \sum_{i=0}^{n-1} \lambda_i * 2 \cdot \vec{v}_i^\top \cdot U \cdot A \cdot A^\top \cdot (E + t \cdot U)^\top \cdot \vec{v}_i$
$g''(t) = \sum_{i=0}^{n-1} \lambda_i * 2 \cdot \vec{v}_i^\top \cdot U \cdot A \cdot A^\top \cdot U^\top \cdot \vec{v}_i$
Since $U \cdot A \cdot A^\top \cdot U^\top$ is positive semi-definite, the second derivative of $g(t)$ is positive $\forall t$. Therefore g is convex, and according to theorem 2, f is convex as well. □

Claim 2.2. *The objective function of the genes inference problem (Methods) is convex (and since it is a minimization problem, it is a convex optimization problem).*

$f(D) = \sum_{i=0}^{n-1} \lambda_i * \vec{v}_i^T * (A * D) * (A * D)^T * \vec{v}_i - \alpha * \|D\|_{L_1}$
$s.t \quad D \quad is \quad diagonal$
$0 \leq D \leq I$

Proof. The set itself is convex (linear inequality). Similarly to claim 2.1, we will define $g(t) = f(E + tU)$, where $E + tU \in \mathbf{dom} f$, and calculate the first and second derivatives:
$g'(t) = \sum_{i=0}^{n-1} \lambda_i * 2 \cdot v^\top \cdot A \cdot U \cdot (D + t \cdot U)^\top \cdot A^\top \cdot v - alpha \cdot \mathrm{tr}(U \cdot \mathrm{sign}(D + t \cdot U)^\top)$
$g''(t) = \sum_{i=0}^{n-1} \lambda_i * 2 \cdot \vec{v}_i^\top \cdot U \cdot A \cdot A^\top \cdot U^\top \cdot \vec{v}_i$
Since $U \cdot A \cdot A^\top \cdot U^\top$ is positive semi-definite, the second derivative of $g(t)$ is positive $\forall t$. Therefore, g is convex, and according to theorem 2, f is convex as well.

□

Claim 2.3. *The objective function of the enhancement and filtering problems (Methods) is convex (and since the filtering problem is a minimization problem, it is a convex optimization problem).*
$f(F) = \sum_{i=0}^{n-1} \lambda_i * \vec{v}_i^T * (A \odot F) * (A \odot F)^T * \vec{v}_i - \alpha * \|F\|$
$s.t \quad 0 \leq F_{i,j} \leq 1 \quad \forall \quad i, j$

Proof. Under the assumption of A being a gene expression matrix with positive entries, we will transform the objective and the set to be:
$f(\tilde{F}) = g(\tilde{F} = \sum_{i=0}^{n-1} \lambda_i * \vec{v}_i^T * \tilde{F} * \tilde{F}^T * \vec{v}_i - \alpha * \sum_{i=0}^{n-1} \sum_{j=0}^{p-1} |\tilde{F}_{i,j}/A_{i,j}^2|)$
$s.t \quad 0 \leq \tilde{F}_{i,j} \leq A_{i,j} \quad \forall \quad i, j,$
where $\tilde{F} = F \odot A$. After the rewriting, we will define $f(\tilde{F}) = g(\tilde{F} + t * \tilde{U})$:
$g'(t) = \sum_{i=0}^{n-1} \lambda_i * 2 \cdot v^\top \cdot \tilde{U} \cdot (\tilde{F} + t \cdot \tilde{U})^\top \cdot v - \alpha * \sum_{i=0}^{n-1} \sum_{j=0}^{p-1} \tilde{U}_{i,j}/A_{i,j}^2$
$g''(t) = \sum_{i=0}^{n-1} \lambda_i * 2 \cdot \vec{v}_i^\top \cdot \tilde{U} \cdot \tilde{U}^\top \cdot \vec{v}_i.$
Similarly to the previous claims, since $U \cdot A \cdot A^\top \cdot U^\top$ is positive semi-definite, the second derivative of $g(t)$ is positive $\forall t$. Therefore g is convex, and according to theorem 2, f is convex as well.

□

B Supplementary figures

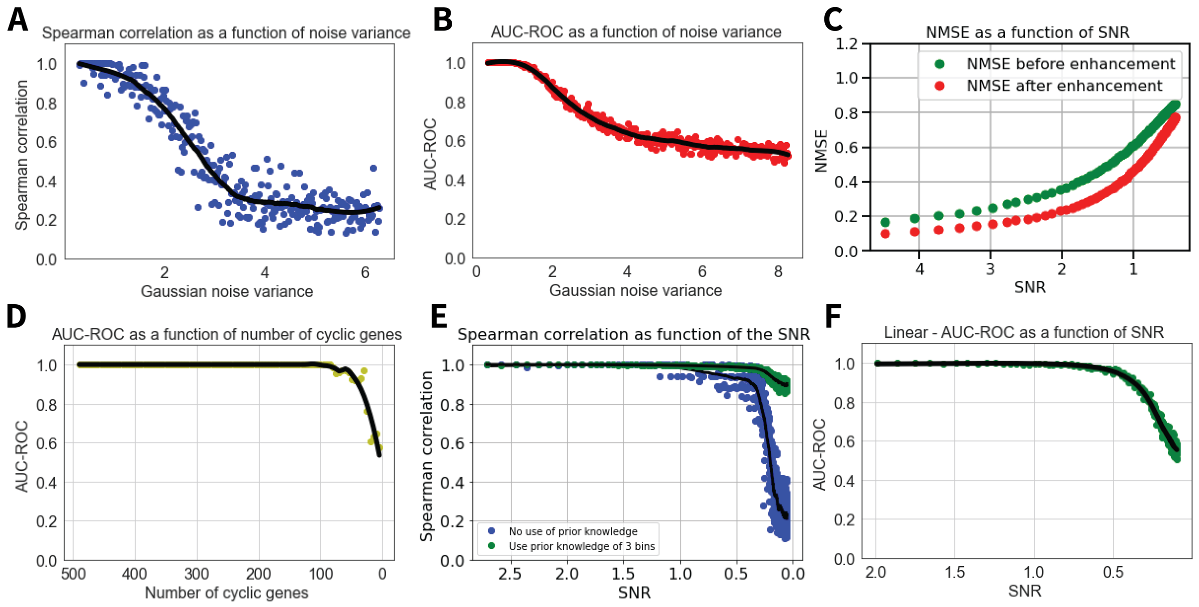

Supplementary Figure 1: (A) Spearman correlation between the ground-truth permutation and the predicted permutation as a function of noise variance over 300 simulations. The reconstruction algorithm was applied to a simulated gene expression matrix based on a cyclic signal of 100 cells and 500 genes, according to the second model. Gaussian noise was added with varying variance. (B) AUC-ROC of informative genes inference task as a function of noise variance over 300 simulation iterations. A cyclic signal and a lineage signal consisting of 256 cells and 250 genes were simulated in each iteration. These signals were concatenated to form a gene expression matrix of 256 cells and 500 genes, and Gaussian noise with varying variances was added to the matrix entry-wise. (C) Shown is NMSE before enhancement ($\frac{\|A-B\|_F}{max(A,B)}$; green), and following enhancement ($\frac{\|A-\tilde{A}\|_F}{max(A,\tilde{A})}$; red), where A is a simulated cyclic signal (according to the spatial model A.2, with 500 genes, 100 cells, and w=0.2), $B = A + N$ (where N is Gaussian noise with variable variance), and $\tilde{A}$ is the enhanced cyclic signal. (D) Applying the reconstruction algorithm and the genes inference algorithm over simulated data with varying number of cyclic genes. In each simulation, 100 cells were simulated with x cyclic genes and $500 - x$ lineage genes. (E) Applying prior knowledge for the reconstruction task. Spearman correlation between the ground-truth permutation and the predicted permutation as a function of SNR. De novo reconstruction (blue), and reconstruction with prior knowledge of low-resolution ordering (3 bins; green), so the mapping of each cell is restricted to the third cycle associated with its label. (F) AUC-ROC of genes inference algorithm as function of noise variance/SNR for inferring linear genes, using the spectrum that is described in the methods section.

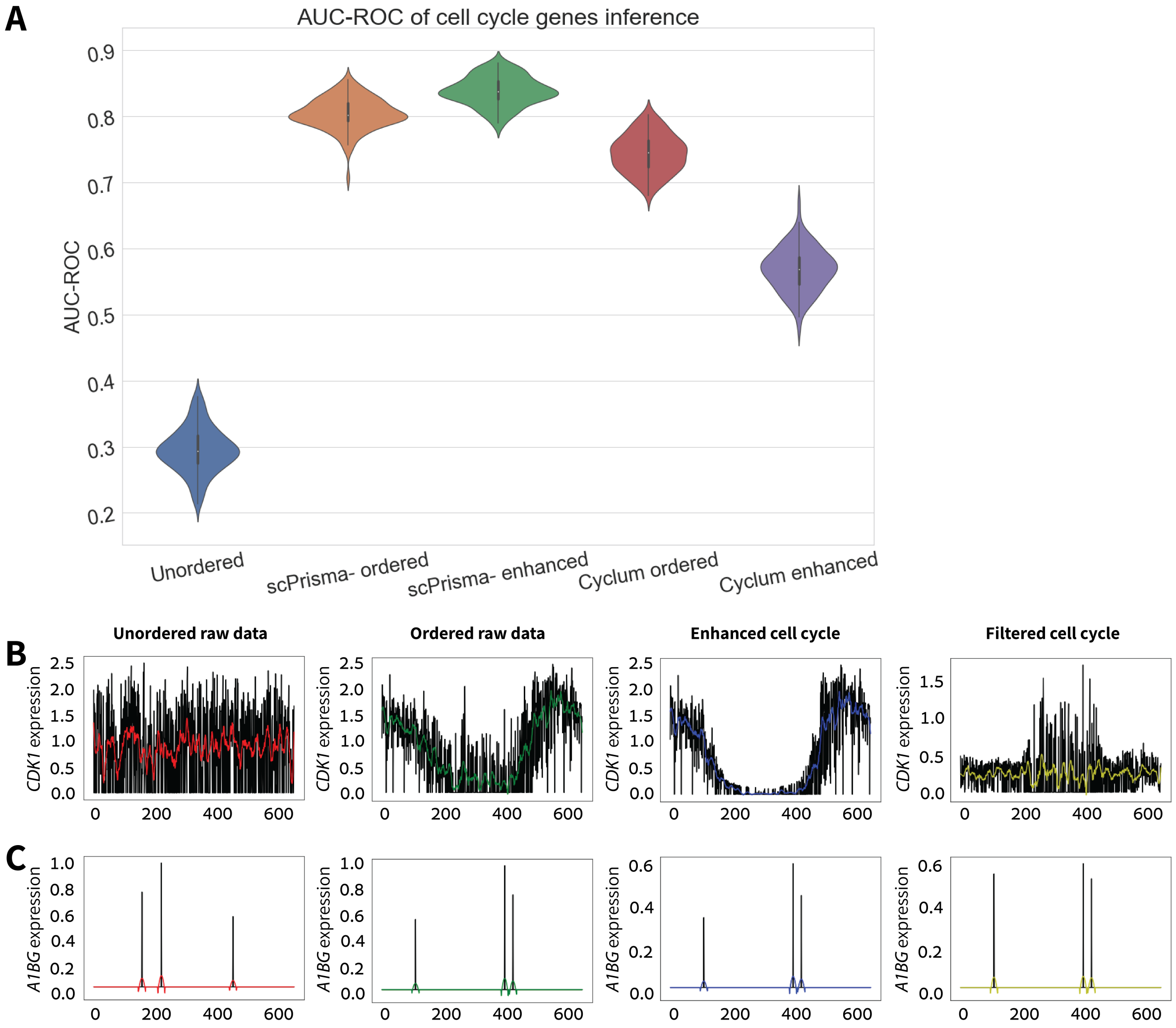

Supplementary Figure 2: Manipulation of HeLa cells data [29] by scPrisma and Cyclum. (A) Gene inference results over extracted circular components in by scPrisma and Cyclum. Violin plot of AUC scores for n=50 experiments of sampling random gene subsets and inference of cyclic genes by scPrisma as cyclic genes based on unordered, ordered (scPrisma and Cyclum), and enhanced (scPrisma and Cyclum) data. The white dot is the median, the gray bar is the interquartile range, and the thin gray line is the rest of the distribution, except for outliers. (B,C) Expression of cell cycle related (B; *CDK1*) and unrelated (C; *A1BG*) genes, manipulated by scPrisma. From left to right: unordered, ordered, enhanced and filtered data.

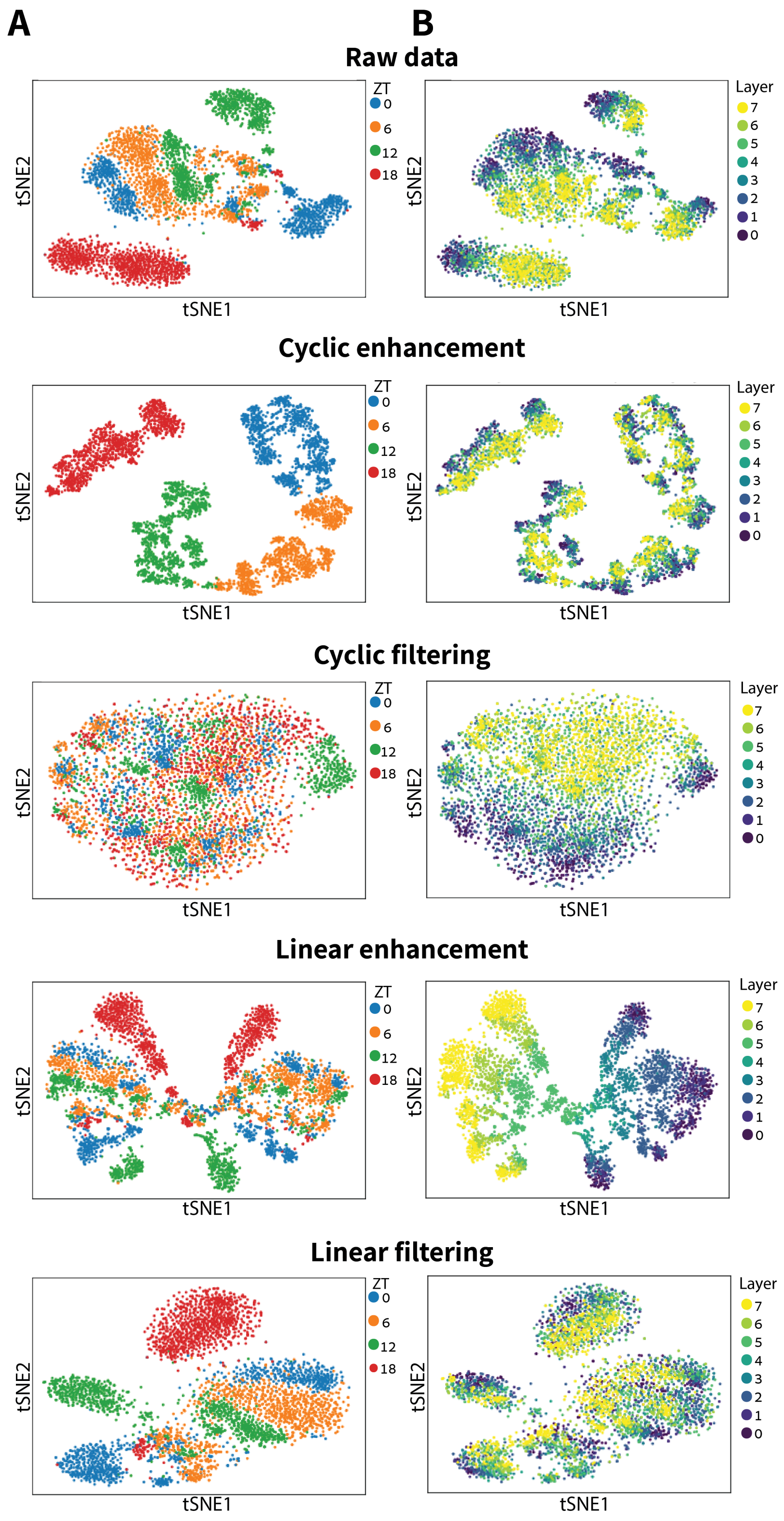

Supplementary Figure 3: Disentanglement of spatial and temporal signals in liver lobules. (A,B) 2D tSNE representation of raw single-cell data, as well as following spectral cyclic enhancement, cyclic filtering, linear enhancement, and linear filtering. The cells are colored either according to their associated time points ('ZT', sampled at four equally-spaced timepoints along the circadian rhythm) (A), or by their respective spatial location ('Layer', according to the zonation analysis done by[6]) (B).

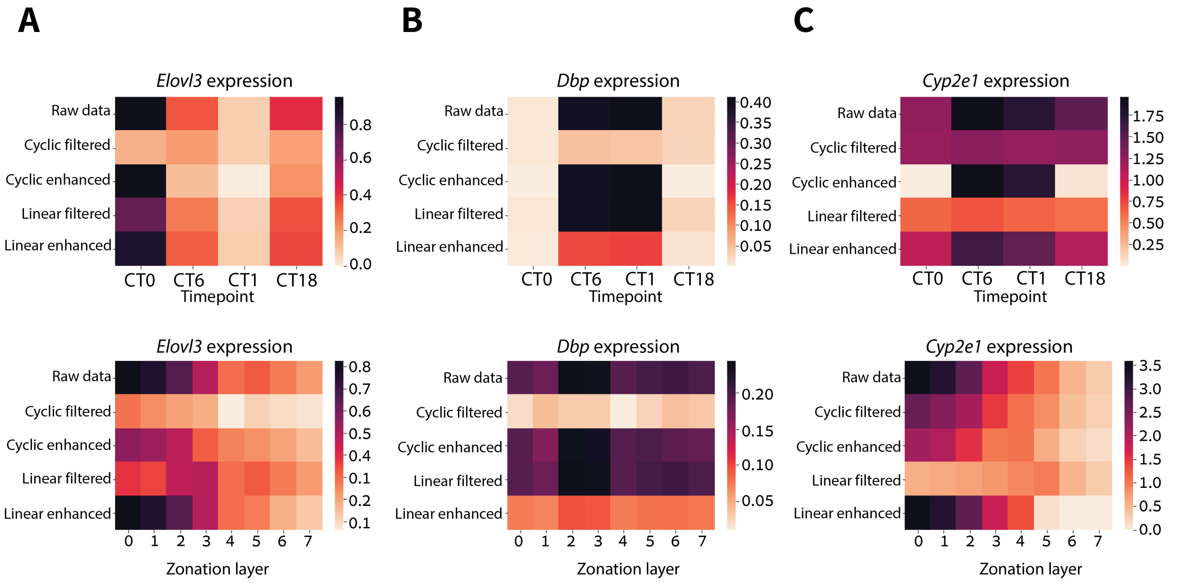

Supplementary Figure 4: Heatmaps of the expression of *Elovl3* (A), *Dbp* (B) and *Cyp2e1* (C) as a function of the sampling time and the zonation layer in liver lobules.

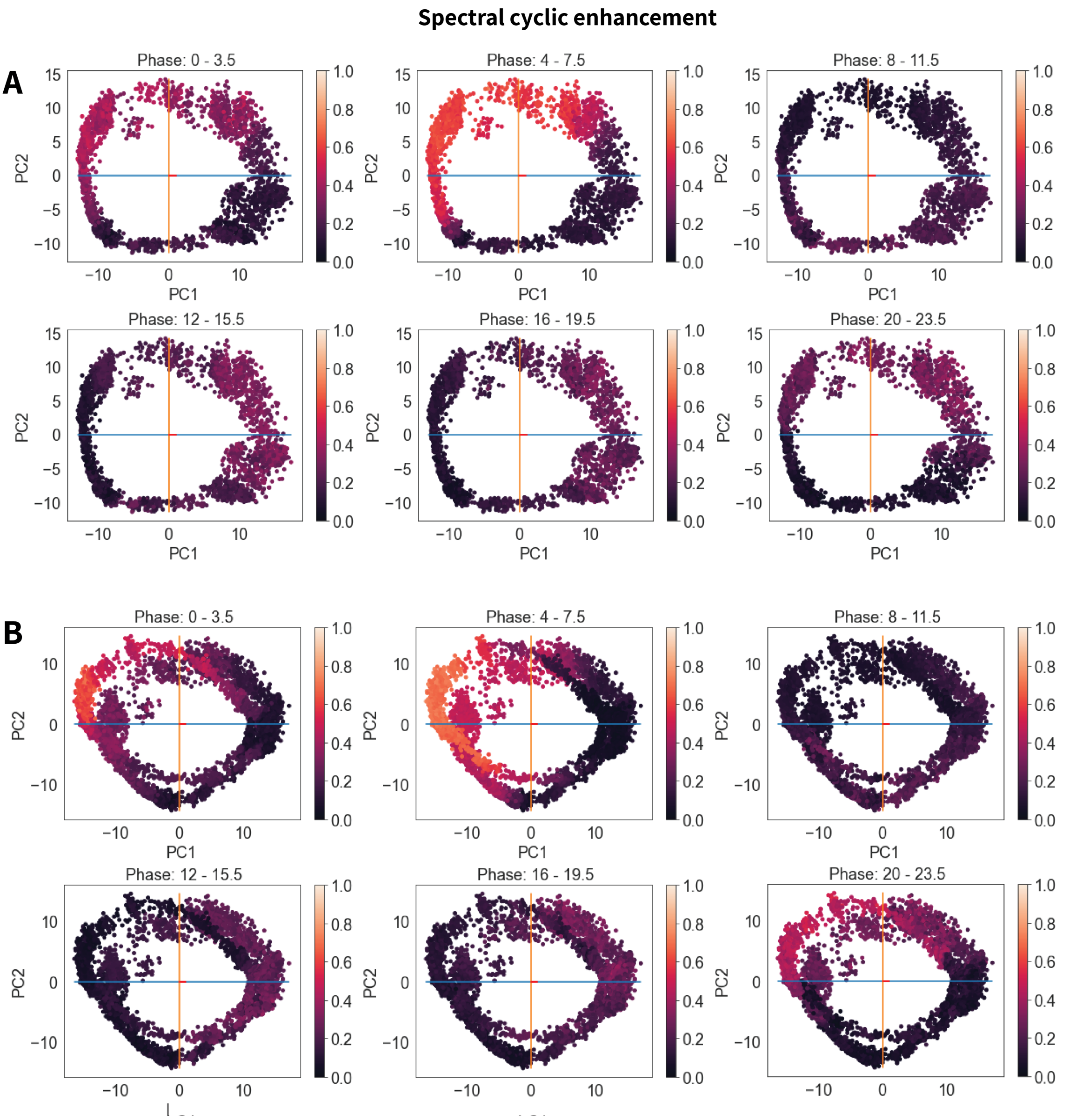

Supplementary Figure 5: Diurnal cycle in Chlamydomonas. (A) 2 dimensional PCA of enhanced cyclic signal of the iron deficient (Fe-) experiment. (B) 2 dimensional PCA of enhanced cyclic signal of the iron deficient (Fe-) data concatenated with the iron replete (Fe+) data. The 24 hours cycle was divided to 6 segments and the coloring reflects the normalized sum of the marker genes that are associated with each phase. The cyclic signal of each one of the experiments was reconstructed separately.

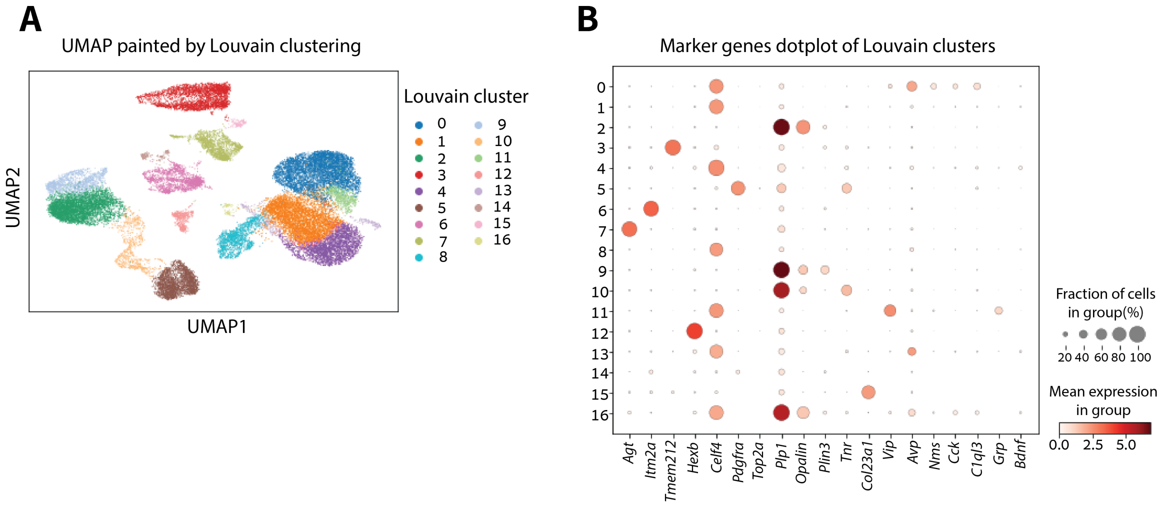

Supplementary Figure 6: Mice suprachiasmatic nucleus clustering. (A) UMAP of the raw SCN data, colored by clusters that were inferred using the Louvain algorithm. (B) Dotplot of the expression of cell type marker genes in each one of the clusters that were inferred using the Louvain algorithm.

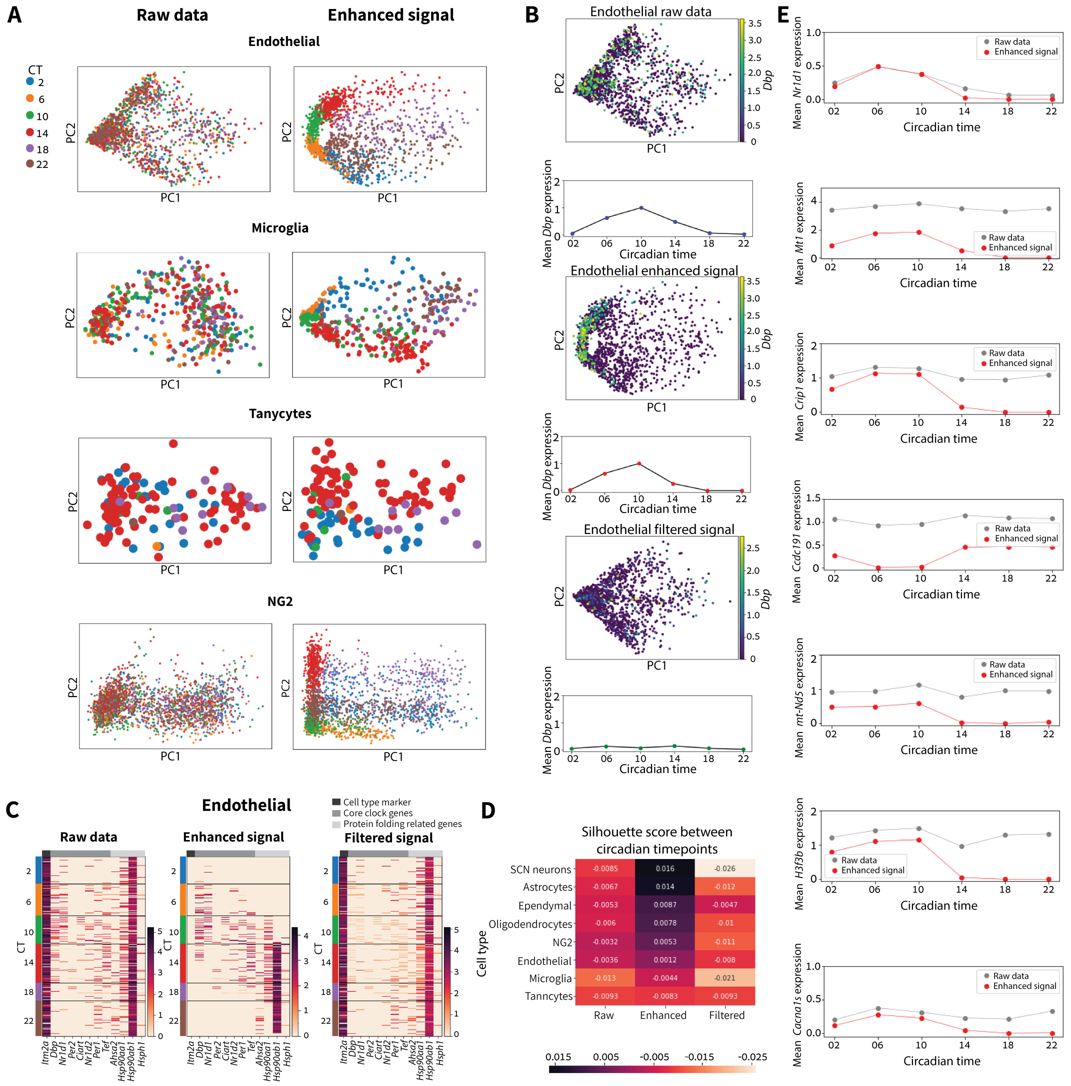

Supplementary Figure 7: Mice suprachiasmatic nucleus disentanglement. (A) 2D PCA of the raw and enhanced data, colored according to the circadian time for several cell types: endothelial, microglia, tancytes and NG2. (B) Mean expression as a function of circadian time of *Dbp*, 2D PCA colored by *Dbp* expression of raw data, enhanced cyclic signal and filtered cyclic signal for endothelial cells. (C) Heatmaps for raw data, enhanced cyclic signal and filtered cyclic signal of expression of (from left to right) cell type marker genes, rhythmic genes and protein folding genes, for endothelial cells. (D) Silhouette score between circadian timepoints for raw data, enhanced cyclic signal and filtered cyclic signal. Cell types with low fraction of circadian genes (according to [19]) exhibit lower scores. (E) Pre- and post-enhancement mean *Nr1d1*, *Mt1*, *Crip1*, *Ccdc191*, *mt-Nd5*, *H3f3b* and *Cacna1s*. expression as a function of circadian time point. Following cyclic enhancement, regulatory interactions between the transcription factor *Nr1d1* and *Mt1*, *Crip1*, *Ccdc191*, *mt-Nd5*, *H3f3b*, *Cacna1s* were uncovered.

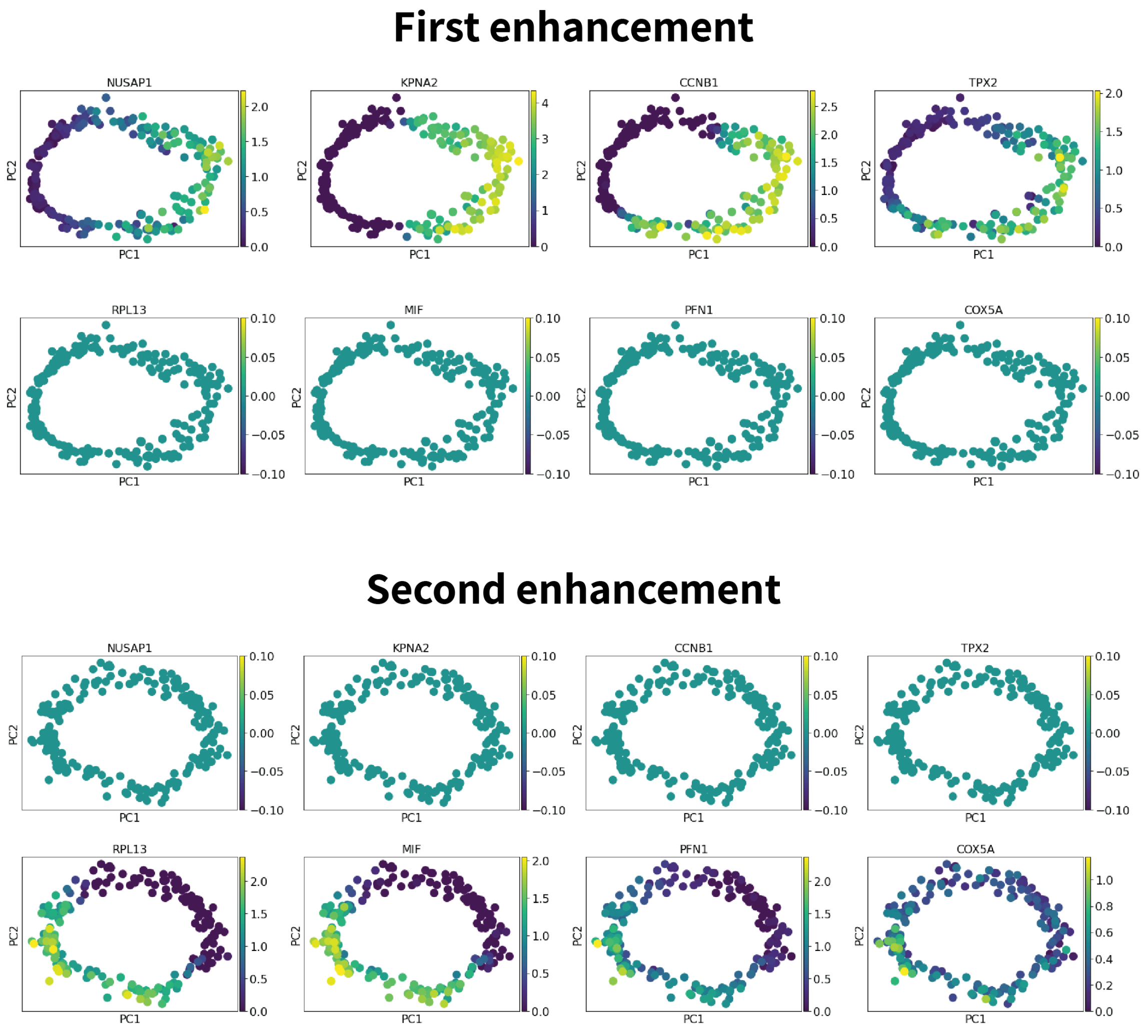

Supplementary Figure 8: Iterative cyclic enhancement by scPrisma of signals in hESC data[16]. (A,B) 2D PCA of enhanced cyclic signal following the cyclic enhancement workflow (reconstruction, gene inference, and enhancement) on the raw hESC data (A), and on the data following cyclic filtering (B). First row in each panel corresponds to cell cycle related marker genes (*NUSAP1, KPNA2, CCNB1, TPX2*), second row corresponds to marker genes associated with an oscillatory pattern related to the experimental setup (chip capture site and output well positions on the Fluidigm C1 chip used for the experiment) (*RPL13, MIF, PFN1, COX5A*).

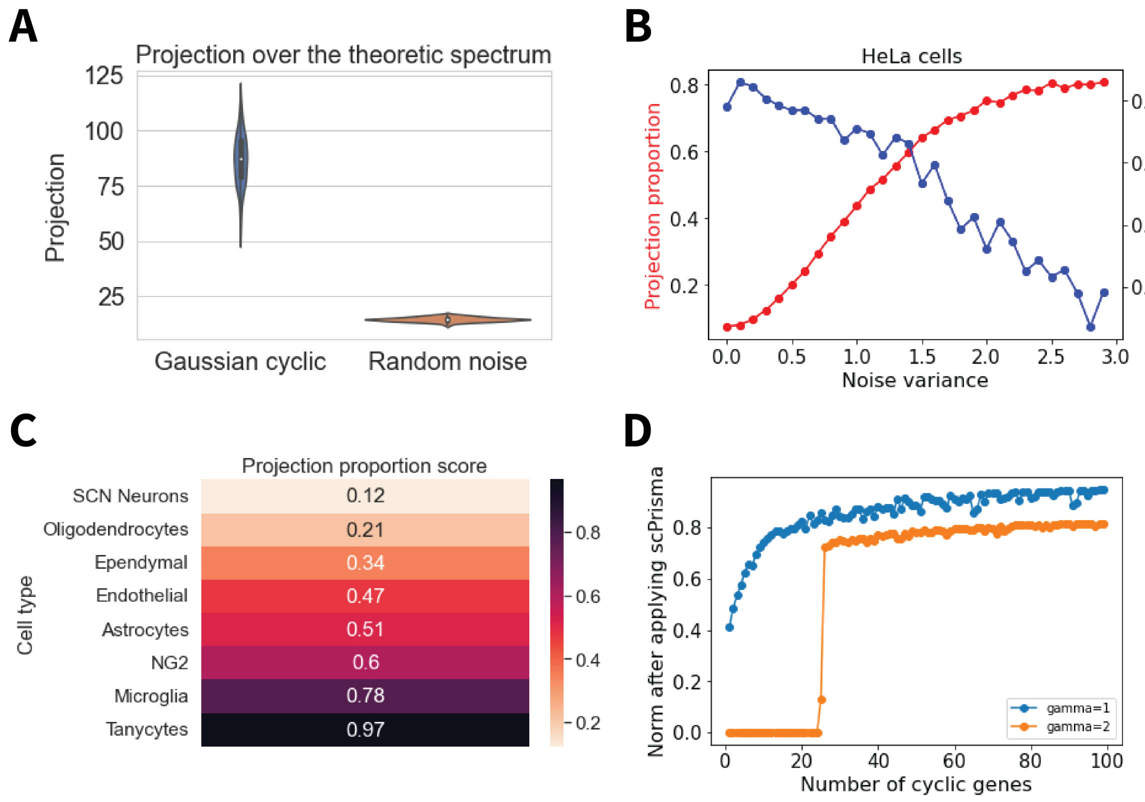

Supplementary Figure 9: (A) Violin plot of the projection of theoretic spectrum of cyclic topology over 100 random simulated cyclic genes (according to model 3, A.2), and 100 random Gaussian noise vectors. Genes were normalized to have unit variance. The white dot is the median, the gray bar is the interquartile range, and the thin gray line is the rest of the distribution, except for outliers. (B) AUC-ROC of cell cycle-related genes inference for HeLa cells (blue; as described in Section 2.2) and projection proportion score (red) as a function of Gaussian noise added to the data. (C) Projection proportion score for different cell types of the SCN data (Section 2.5). (D) Change in L2 entry-wise norm following scPrisma enhancement workflow, for different regularization values of the gene inference task. We simulated 100X100 matrix of random Gaussian noise (mean=0, variance=0.5, the expression matrix was clipped to be positive), and cyclic gene expression matrix of 100 genes and 100 cells (Methods). For every i between 1 and 100, we added 'i' cyclic genes over 'i' noise vectors. Then, concatenated $100 - i$ other noise vectors, normalized the matrix L2 entry-wise norm to 1 and applied the full scPrisma enhancement workflow (Methods). Finally we calculated the L2 entry-wise norm after applying the enhancement workflow for varying number of cyclic genes.

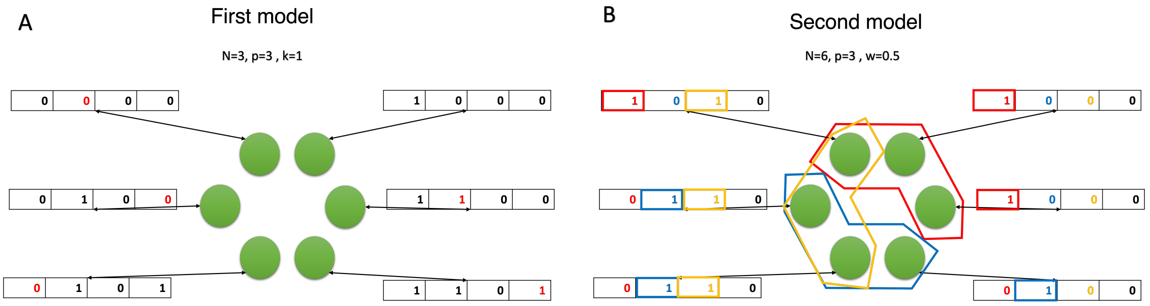

Supplementary Figure 10: Illustration of two cyclic signal models: the progression (A) and spatial (B) models.

Supplementary References

[1] Maayan Baron et al. “A single-cell transcriptomic map of the human and mouse pancreas reveals inter-and intra-cell population structure”. In: *Cell systems* 3.4 (2016), pp. 346–360.

[2] Martin Barron and Jun Li. “Identifying and removing the cell-cycle effect from single-cell RNA-Sequencing data”. In: *Scientific reports* 6.1 (2016), pp. 1–10.

[3] Stephen Boyd, Stephen P Boyd, and Lieven Vandenberghe. *Convex optimization.* Cambridge university press, 2004.

[4] Andrew Butler et al. “Integrating single-cell transcriptomic data across different conditions, technologies, and species”. In: *Nature biotechnology* 36.5 (2018), pp. 411–420.

[5] Tadeusz Caliński and Jerzy Harabasz. “A dendrite method for cluster analysis”. In: *Communications in Statistics-theory and Methods* 3.1 (1974), pp. 1–27.

[6] Colas Droin et al. “Space-time logic of liver gene expression at sub-lobular scale”. In: *Nature Metabolism* 3.1 (2021), pp. 43–58.

[7] Eran Eden et al. “GOrilla: a tool for discovery and visualization of enriched GO terms in ranked gene lists”. In: *BMC bioinformatics* 10.1 (2009), pp. 1–7.

[8] Mirjana Efremova et al. “CellPhoneDB: inferring cell–cell communication from combined expression of multi-subunit ligand–receptor complexes”. In: *Nature protocols* 15.4 (2020), pp. 1484–1506.

[9] Ky Fan. “Maximum properties and inequalities for the eigenvalues of completely continuous operators”. In: *Proceedings of the National Academy of Sciences of the United States of America* 37.11 (1951), p. 760.

[10] Laleh Haghverdi et al. “Batch effects in single-cell RNA-sequencing data are corrected by matching mutual nearest neighbors”. In: *Nature biotechnology* 36.5 (2018), pp. 421–427.

[11] Keren Bahar Halpern et al. “Single-cell spatial reconstruction reveals global division of labour in the mammalian liver”. In: *Nature* 542.7641 (2017), pp. 352–356.

[12] Terry F Hayamizu, Richard A Baldock, and Martin Ringwald. “Mouse anatomy ontologies: enhancements and tools for exploring and integrating biomedical data”. In: *Mammalian Genome* 26.9 (2015), pp. 422–430.

[13] Brian Hie, Bryan Bryson, and Bonnie Berger. “Efficient integration of heterogeneous single-cell transcriptomes using Scanorama”. In: *Nature biotechnology* 37.6 (2019), pp. 685–691.

[14] S Rao Jammalamadaka and Ambar Sengupta. *Topics in circular statistics.* Vol. 5. world scientific, 2001.

[15] Ilya Korsunsky et al. “Fast, sensitive and accurate integration of single-cell data with Harmony”. In: *Nature methods* 16.12 (2019), pp. 1289–1296.

[16] Ning Leng et al. “Oscope identifies oscillatory genes in unsynchronized single-cell RNA-seq experiments”. In: *Nature methods* 12.10 (2015), pp. 947–950.

[17] Shaoheng Liang et al. “Latent periodic process inference from single-cell RNA-seq data”. In: *Nature communications* 11.1 (2020), pp. 1–8.

[18] Mohammad Lotfollahi, F Alexander Wolf, and Fabian J Theis. “scGen predicts single-cell perturbation responses”. In: *Nature methods* 16.8 (2019), pp. 715–721.

[19] Danyi Ma et al. “Spatiotemporal single-cell analysis of gene expression in the mouse suprachiasmatic nucleus”. In: *Nature neuroscience* 23.3 (2020), pp. 456–467.

[20] Feiyang Ma et al. “Single-cell RNA sequencing of batch Chlamydomonas cultures reveals heterogeneity in their diurnal cycle phase”. In: *The Plant Cell* 33.4 (2021), pp. 1042–1057.

[21] Thomas Moerman et al. “GRNBoost2 and Arboreto: efficient and scalable inference of gene regulatory networks”. In: *Bioinformatics* 35.12 (2019), pp. 2159–2161.

[22] Mauro J Muraro et al. “A single-cell transcriptome atlas of the human pancreas”. In: *Cell systems* 3.4 (2016), pp. 385–394.

[23] Mor Nitzan and Michael P Brenner. “Revealing lineage-related signals in single-cell gene expression using random matrix theory”. In: *Proceedings of the National Academy of Sciences* 118.11 (2021).

[24] Katsutaka Oishi. "Plasminogen activator inhibitor-1 and the circadian clock in metabolic disorders". In: *Clinical and experimental hypertension* 31.3 (2009), pp. 208–219.

[25] Daisuke Ono, Ken-ichi Honma, and Sato Honma. "Roles of Neuropeptides, VIP and AVP, in the Mammalian Central Circadian Clock". In: *Frontiers in neuroscience* 15 (2021), p. 351.

[26] Giovanni Palla et al. "Squidpy: a scalable framework for spatial omics analysis". In: *Nature methods* 19.2 (2022), pp. 171–178.

[27] Chongli Qin and Lucy J Colwell. "Power law tails in phylogenetic systems". In: *Proceedings of the National Academy of Sciences* 115.4 (2018), pp. 690–695.

[28] Rahul Satija et al. "Spatial reconstruction of single-cell gene expression data". In: *Nature biotechnology* 33.5 (2015), pp. 495–502.

[29] Daniel Schwabe et al. "The transcriptome dynamics of single cells during the cell cycle". In: *Molecular systems biology* 16.11 (2020), e9946.

[30] Åsa Segerstolpe et al. "Single-cell transcriptome profiling of human pancreatic islets in health and type 2 diabetes". In: *Cell metabolism* 24.4 (2016), pp. 593–607.

[31] Robert R Stickels et al. "Highly sensitive spatial transcriptomics at near-cellular resolution with Slide-seqV2". In: *Nature biotechnology* 39.3 (2021), pp. 313–319.

[32] Daniela Strenkert et al. "Multiomics resolution of molecular events during a day in the life of Chlamydomonas". In: *Proceedings of the National Academy of Sciences* 116.6 (2019), pp. 2374–2383.

[33] Tim Stuart et al. "Comprehensive integration of single-cell data". In: *Cell* 177.7 (2019), pp. 1888–1902.

[34] Fei Wang, Ping Li, and Arnd Christian Konig. "Learning a bi-stochastic data similarity matrix". In: *2010 IEEE International Conference on Data Mining*. IEEE. 2010, pp. 551–560.

[35] Yue J Wang et al. "Single-cell transcriptomics of the human endocrine pancreas". In: *Diabetes* 65.10 (2016), pp. 3028–3038.

[36] Eric E Zhang and Steve A Kay. "Clocks not winding down: unravelling circadian networks". In: *Nature reviews Molecular cell biology* 11.11 (2010), pp. 764–776.

[37] Grace XY Zheng et al. "Massively parallel digital transcriptional profiling of single cells". In: *Nature communications* 8.1 (2017), pp. 1–12.
