# Supplementary material for: scPrisma infers, filters and enhances topological signals in single-cell data using spectral template matching
Source: Nat Biotechnol. 2023 Feb 27;41(11):1645–54. doi: 10.1038/s41587-023-01663-5 (PMC10635821; doi:10.1038/s41587-023-01663-5)
Supplement: Supplementary file 2 — Reporting Summary [file 41587_2023_1663_MOESM2_ESM.pdf]

nature portfolio

Corresponding author(s): Mor Nitzan

Last updated by author(s): Jan 2, 2023

Reporting Summary

Nature Portfolio wishes to improve the reproducibility of the work that we publish. This form provides structure for consistency and transparency in reporting. For further information on Nature Portfolio policies, see our Editorial Policies and the Editorial Policy Checklist.

Statistics

For all statistical analyses, confirm that the following items are present in the figure legend, table legend, main text, or Methods section.

n/a	Confirmed	
☐	☒	The exact sample size (*n*) for each experimental group/condition, given as a discrete number and unit of measurement
☒	☐	A statement on whether measurements were taken from distinct samples or whether the same sample was measured repeatedly
☐	☒	The statistical test(s) used AND whether they are one- or two-sided<br>*Only common tests should be described solely by name; describe more complex techniques in the Methods section.*
☒	☐	A description of all covariates tested
☒	☐	A description of any assumptions or corrections, such as tests of normality and adjustment for multiple comparisons
☐	☒	A full description of the statistical parameters including central tendency (e.g. means) or other basic estimates (e.g. regression coefficient) AND variation (e.g. standard deviation) or associated estimates of uncertainty (e.g. confidence intervals)
☐	☒	For null hypothesis testing, the test statistic (e.g. *F*, *t*, *r*) with confidence intervals, effect sizes, degrees of freedom and *P* value noted<br>*Give P values as exact values whenever suitable.*
☒	☐	For Bayesian analysis, information on the choice of priors and Markov chain Monte Carlo settings
☒	☐	For hierarchical and complex designs, identification of the appropriate level for tests and full reporting of outcomes
☒	☐	Estimates of effect sizes (e.g. Cohen's *d*, Pearson's *r*), indicating how they were calculated

Our web collection on statistics for biologists contains articles on many of the points above.

Software and code

Policy information about availability of computer code

Data collection

Data analysis

Our code is publicly available at:
https://github.com/nitzanlab/scPrisma/

We used the following python packages:
Scanpy (version 1.9.1)
Squidpy (version 1.2.2)
Louvain (version 0.7.1)
Scipy (version 1.9.3)
Leidenalg (version 0.8.10)
Numba (version 0.55.2)
Numpy (version 1.22.0)
Torch (version 1.12.1)
GRNBoost2 (arboreto package version: 0.1.6)

For manuscripts utilizing custom algorithms or software that are central to the research but not yet described in published literature, software must be made available to editors and reviewers. We strongly encourage code deposition in a community repository (e.g. GitHub). See the Nature Portfolio guidelines for submitting code & software for further information.

Data

Policy information about availability of data

All manuscripts must include a data availability statement. This statement should provide the following information, where applicable:

- Accession codes, unique identifiers, or web links for publicly available datasets
- A description of any restrictions on data availability
- For clinical datasets or third party data, please ensure that the statement adheres to our policy

The scRNA-seq datasets used for this study were acquired from the Gene Expression Omnibus (GEO) database with the following accession numbers: HeLaS3 (GSM4224315), liver (GSE145197), Chlamydomonas (GSE157580) and SCN (GSE117295).
The slide-seq v2 data is available on the Broad Institute website : https://singlecell.broadinstitute.org/single_cell/study/SCP815/highly-sensitive-spatial-transcriptomics-at-near-cellular-resolution-with-slide-seqv2#study-download
The pancreas data is available on: ftp://ngs.sanger.ac.uk/production/teichmann/BBKNN/objects-pancreas.zip
The slide-seq v2 data was collected using Squidpy (version 1.2.2).
The pancreas data was collected using Scanpy (version 1.9.1).
List of genes:
Cell cycle genes: https://www.embopress.org/doi/full/10.15252/msb.20209946
Diurnal cycle genes: https://www.pnas.org/doi/10.1073/pnas.1815238116
Mus musculus transcription factors list (for gene regulatory network inference): http://bioinfo.life.hust.edu.cn/AnimalTFDB/
Ligand-receptor interactions (CellPhoneDB)- https://www.nature.com/articles/s41596-020-0292-x

Human research participants

Policy information about studies involving human research participants and Sex and Gender in Research.

Reporting on sex and gender	N/A
Population characteristics	N/A
Recruitment	N/A
Ethics oversight	N/A

Note that full information on the approval of the study protocol must also be provided in the manuscript.

Field-specific reporting

Please select the one below that is the best fit for your research. If you are not sure, read the appropriate sections before making your selection.

☒ Life sciences ☐ Behavioural & social sciences ☐ Ecological, evolutionary & environmental sciences

For a reference copy of the document with all sections, see nature.com/documents/nr-reporting-summary-flat.pdf

Life sciences study design

All studies must disclose on these points even when the disclosure is negative.

Sample size

No experimental data was produced as part of this study.
Figure 2F and Supplementary Figure 11A, the sample size is n=50. Extended data figure 1E, the sample size is n=100.

Data exclusions

HeLa cells:
As recommended in the original study, we filtered out cells with low number of counts, until the mean counts reached 4,500 UMI counts per cell.
Liver:
We randomly sampled 1,000 cells from each timepoint (500 from each batch, for having the same number of cells from each time point).
Chlamydomonas:
We randomly sampled 3,000 cells from each condition (for having the same number of cells at each condition, and because of the runtime complexity of ccRemover).
SCN:
The neural population analysis was concentrated on the SCN neurons, as they exhibit clear temporal peaks of the core clock genes in contrast to nearly flat expression of these genes in the remaining neurons.
Slide-Seq data:
We randomly sampled 70% of the cells (29250/41786) because of memory consumption considerations and the runtime of the all shortest

	paths algorithms.
Replication	No experimental data was produced as part of this study.
Randomization	No experimental data was produced as part of this study.
Blinding	No experimental data was produced as part of this study.

Reporting for specific materials, systems and methods

We require information from authors about some types of materials, experimental systems and methods used in many studies. Here, indicate whether each material, system or method listed is relevant to your study. If you are not sure if a list item applies to your research, read the appropriate section before selecting a response.

Materials & experimental systems

n/a	Involved in the study
☒	☐ Antibodies
☒	☐ Eukaryotic cell lines
☒	☐ Palaeontology and archaeology
☒	☐ Animals and other organisms
☒	☐ Clinical data
☒	☐ Dual use research of concern

Methods

n/a	Involved in the study
☒	☐ ChIP-seq
☒	☐ Flow cytometry
☒	☐ MRI-based neuroimaging
